# CD4^+^T cells mediate protection against Zika associated severe disease in a mouse model of infection

**DOI:** 10.1371/journal.ppat.1007237

**Published:** 2018-09-13

**Authors:** Mariah Hassert, Kyle J. Wolf, Katherine E. Schwetye, Richard J. DiPaolo, James D. Brien, Amelia K. Pinto

**Affiliations:** 1 Department of Molecular Microbiology and Immunology, Saint Louis University, St Louis, Missouri, United States of America; 2 Department of Pathology, Saint Louis University, St. Louis, Missouri, United States of America; Icahn School of Medicine at Mount Sinai, UNITED STATES

## Abstract

Zika virus (ZIKV) has gained worldwide attention since it emerged, and a global effort is underway to understand the correlates of protection and develop diagnostics to identify rates of infection. As new therapeutics and vaccine approaches are evaluated in clinical trials, additional effort is focused on identifying the adaptive immune correlates of protection against ZIKV disease. To aid in this endeavor we have begun to dissect the role of CD4^+^T cells in the protection against neuroinvasive ZIKV disease. We have identified an important role for CD4^+^T cells in protection, demonstrating that in the absence of CD4^+^T cells mice have more severe neurological sequela and significant increases in viral titers in the central nervous system (CNS). The transfer of CD4^+^T cells from ZIKV immune mice protect type I interferon receptor deficient animals from a lethal challenge; showing that the CD4^+^T cell response is necessary and sufficient for control of ZIKV disease. Using a peptide library spanning the complete ZIKV polyprotein, we identified both ZIKV-encoded CD4^+^T cell epitopes that initiate immune responses, and ZIKV specific CD4^+^T cell receptors that recognize these epitopes. Within the ZIKV antigen-specific TCRβ repertoire, we uncovered a high degree of diversity both in response to a single epitope and among different mice responding to a CD4^+^T cell epitope. Overall this study identifies a novel role for polyfunctional and polyclonal CD4^+^T cells in providing protection against ZIKV infection and highlights the need for vaccines to develop robust CD4^+^T cell responses to prevent ZIKV neuroinvasion and limit replication within the CNS.

## Author summary

Since the detection of ZIKV in the Americas in 2015, urgent efforts have been made to develop ZIKV-specific methods of diagnosis and novel vaccines. However, a full understanding of the natural history of infection, interactions with other arboviruses, and the protection mediated by therapeutics or vaccination is hindered by our limited means of assessing ZIKV-specific immune responses. In this study, we investigated the role of CD4^+^T cells in protection from ZIKV using a mouse model of infection. We found that CD4^+^T cells are both necessary and sufficient for protection from ZIKV severe disease. We have identified the ZIKV-encoded CD4^+^T cell epitopes which are targeted during infection and the cognate T cell receptors to these epitopes. This study underscores the need to develop ZIKV vaccines which elicit strong CD4^+^T cell responses to potentially provide protection from ZIKV neuroinvasion and replication in the CNS.

## Introduction

As a member of the family Flaviviridae, Zika virus (ZIKV) is related to mosquito spread arboviruses including West Nile virus (WNV), dengue virus (DENV), Japanese encephalitis virus (JEV), and yellow fever virus (YFV). While having been identified in 1947 [1], ZIKV first entered the dialogue of emerging pathogens following a large outbreak on Yap island in the Federated States of Micronesia in 2007 [2]. This unexpected emergence of ZIKV beyond its traditional borders and the subsequent global spread of the virus in the last ten years (Reviewed in [3]) has caused a public health emergency. As ZIKV spread, infection has become associated with increased risks for congenital birth defects and neurological disease [4–12]. As it is now recognized that there is an increased risk of severe disease associated with ZIKV infection, focus has shifted toward detection, defining correlates of protection, and the development of a vaccine or antiviral to protect against disease progression.

ZIKV is a single stranded positive sense RNA virus that shares a high degree of similarity both structurally and genetically with other flaviviruses including the co-circulating DENVs. The 11kb genome is translated as a single polypeptide that is post-translationally processed by cellular and viral proteases into ten viral proteins; three structural proteins, pre/membrane (M/prM,) Capsid (C), and Envelope (E), and seven nonstructural proteins, (NS1, NS2a, NS2b, NS3, NS4a, NS4b and NS5) [13]. A benefit of the high degree of similarity between ZIKV and related flaviviruses is that many of the tools and techniques that have been established with related flaviviruses are readily adaptable to study ZIKV and this has allowed many flavivirus laboratories to readily adapt to studying this emerging pathogen leading to a rapid understanding of disease pathogenesis. However, from a public health perspective, this high degree of similarity between flaviviruses is a significant disadvantage, as it is very difficult to determine if an individual is infected with ZIKV or a related flavivirus as the symptoms of disease are often very similar and structural similarities with co-circulating flaviviruses confound most rapid serological tests [14–19]. Previous studies have shown that the antibody response generated against one flavivirus is able to bind to a heterologous flavivirus [20–22] making it difficult to use a traditional serological test to identify a ZIKV infection. With the rapid spread and ZIKV associated disease severity in flavivirus endemic areas, knowing the correlates of protection that are cross-reactive and specific to ZIKV has taken on a higher level of importance.

While ZIKV shares many features with related flaviviruses, differences in diseases, viral persistence and viral transmission between ZIKV and other flaviviruses suggest that the immune response and the correlates of protection may be unique. Studies looking at both human and nonhuman primate infections with ZIKV have identified a strong adaptive immune response [23] with detectable humoral and cellular immune responses. Previous studies have shown roles for the innate immune response as well as antibodies and T cells in protecting susceptible small animal models against lethal ZIKV infection [20, 23–32]. These studies point to a multifaceted response with both cellular and humoral immunity playing a role in protecting against ZIKV infection.

In this study, we focus on the ZIKV-reactive CD4^+^T cell response in a mouse model of infection. CD4^+^T cells have been shown to be important for controlling some flavivirus infections [33–38], but a role for CD4^+^T cells during ZIKV infection is still unclear. Reports have identified the presence of CD4^+^T cells during ZIKV infection but their function during the course of infection has yet to be determined. To determine if CD4^+^T cells were protective against ZIKV infection we utilized the Ifnar1^-/-^ mouse model of infection. ZIKV has been shown to inhibit type I interferon receptor (Ifnar) signaling through the inhibition of STAT2 in humans [39]. To mimic this inhibition in mice we have used the type 1 interferon receptor deficient mice (Ifnar1^-/-^), that have previously been shown to be susceptible to infection by ZIKV [26]. Previous studies have shown that the Ifnar1^-/-^ mouse is a good model for establishing correlates [26, 40] of protection as well as early efficacy testing of ZIKV therapeutics and vaccines. Using the Ifnar1^-/-^ mouse, our results indicate that in the absence of CD4^+^T cells there is a high level of morbidity and mortality as well as increases in paralysis and tremors in the CD4 deficient ZIKV infected mice, indicating that CD4^+^T cells are necessary for the protection against a lethal ZIKV challenge. Our results also show that adoptively transferred CD4^+^T cells from ZIKV immunized mice protected susceptible mice from a lethal ZIKV challenge. Once we determined that CD4^+^T cells were protective, we performed a screen with an overlapping peptide library covering the ZIKV proteins and identified multiple epitopes that stimulated CD4^+^T cell responses during ZIKV infection. Characterization of these ZIKV specific CD4^+^T cells revealed that they were both polyfunctional in their responses and polyclonal in the T cell receptors used to recognize ZIKV epitopes. Overall these data demonstrate that generating polyfunctional and polyclonal CD4^+^T cell responses are important correlates for protection during ZIKV infection.

## Results

### CD4^+^T cells are necessary for the protection against a lethal ZIKV challenge

**Fig 1:**
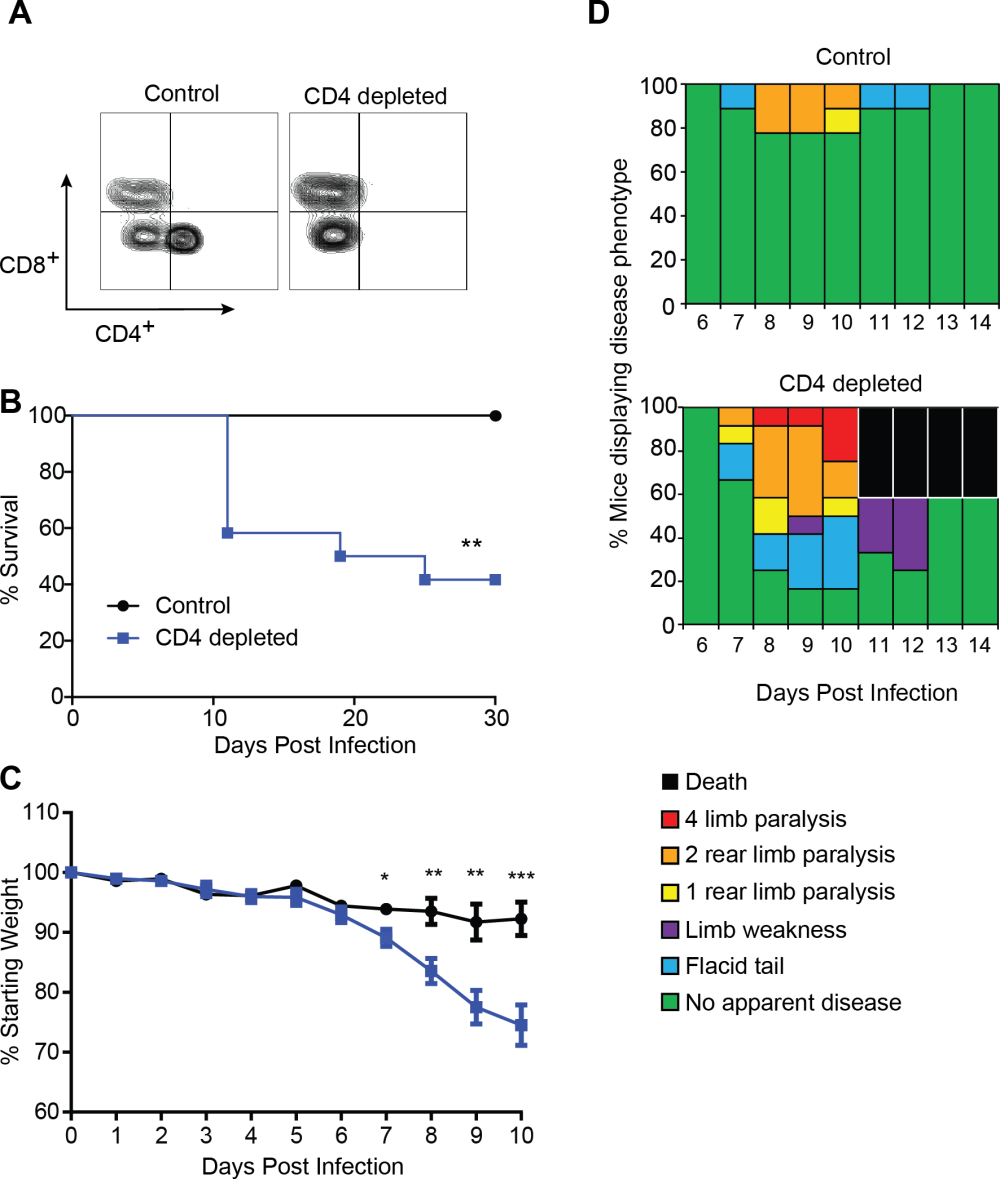
CD4^+^T cells are necessary for protection from ZIKV challenge. **(A)** Confirmation of CD4^+^T cell depletion. On day –3 and day 0, mice were administered 100 μg of depleting antibody anti-CD4 or isotype control intraperitoneally (n = 9 or 12 mice per group respectively). Blood was harvested at the time of infection and the cells were stained for CD3, CD19, CD4, and CD8. Cells were gated by a lymphocyte gate, CD3^+^, CD19^-^, and CD4^+^ or CD8^+^. Greater than 99% depletion of CD4^+^T cells was achieved. **(B)** Survival of ten- to twelve-week-old Ifnar1^-/-^ mice following CD4^+^T cell depletion and inoculation with 10^5^ FFU of ZIKV by footpad injection. (n = 9 control, n = 12 depleted). Survival differences were statistically significant (**, p = 0.0063) as determined using a Mantel-Cox test. **(C)** Weight loss during acute ZIKV infection of ten- to twelve-week-old mice. As a measure of disease, mice were weighed daily for 10 days. On days 7 (*, p = 0.011), 8 (**, p = 0.0039), 9 (**, p = 0.0029), and 10 (***, p = 0.0007) mice from the CD4^+^ depleted group lost significantly more weight than the control group as determined using an unpaired t test with Welch's correction. **(D)** Neurological sequela associated with acute ZIKV infection. Mice were evaluated for signs of neurological disease daily and graphed on each day as a percentage of mice displaying that disease indicator. Signs of disease range and in the most severe cases accelerate in the following manner from no apparent disease, limp tail, hind limb weakness, hind limb paralysis, complete paralysis and death. All data is a compellation of 2 independent experiments.

We determined the importance of CD4^+^T cell responses in protecting against ZIKV disease by first depleting CD4^+^T cell from 10-12-week-old Ifnar deficient (Ifnar1^-/-^) mice. The Ifnar1^-/-^ mice received the CD4^+^T cell depleting antibody three days prior to infection and a second dose on the day of subcutaneous (SC) infection with 1x10^5^ focus forming units (FFU) of ZIKV. CD4^+^T cell depletion was confirmed by flow cytometry analyses of blood on all of the mice (n = 12) on day 4 post infection and it was determined that less than 1% of the CD4^+^T cells remained in mice from the depleted group (Fig 1A). Following ZIKV infection we monitored the mice daily, recording: mortality, weight, and clinical signs of disease (Fig 1B–1D). In both the CD4 depleted and control mice we saw evidence of ZIKV infection and disease, which included weight loss, and temporary hind limb paralysis beginning day 7 post infection.

We noted a significant difference in the mortality between the CD4 depleted and control mice, where 60 percent of the depleted mice succumb to the infection compared to 100 percent survival of the control Ifnar1^-/-^ animals (Fig 1B). In the Ifnar1^-/-^ mice the difference in mortality corresponded to a significant weight loss in CD4 depleted versus control animals (Fig 1C) and more severe clinical signs of disease (Fig 1D) during the course of infection. Seven days after infection, the CD4^+^T cell-depleted Ifnar1^-/-^ mice showed a significant difference in weight that was maintained until day eleven post infection, when the CD4 depleted mice began to succumb to infection. We also monitored the mice during infection and evaluated their disease state until day fourteen post infection (Fig 1D). The CD4 depleted mice showed elevated signs of neurological disease compared to the control mice beginning on day seven. By day 12 post infection the control mice had either succumb to infection or no longer showed signs of clinical disease (Fig 1D). While some mice in both the depleted and control groups demonstrated some form of hind limb weakness that was first seen on day seven post infection (Fig 1D), we noted whole body tremors and muscle shaking uniquely in the ZIKV infected CD4-depleted mice that we did not observe in the control group. We also noted that only CD4 depleted mice with hind limb paralysis were completely unresponsive to hind limb pressure applied with forceps. These findings suggested exacerbated nerve or muscle involvement in the CD4 depleted mice compared to the control group. To determine if the CD4 depleted mice that were found to display these signs of neurological disease had more severe CNS inflammation than the non-depleted mice, which in general displayed only moderate clinical signs of disease, we performed hematoxylin and eosin (H&E) staining on sections of the brains of depleted and control mice at day 8 post-infection (n = 4–5 per group) (S1 Fig). We found that both the control (S1A Fig) and CD4 depleted (S1B Fig) groups both displayed some amount of inflammation relative to an uninfected control (S1C Fig). In general, the CD4 depleted group was characterized by moderate to severe perineuronal inflammation with suspected microglial nodules and perivascular inflammation with inflammation in the leptomeninges, cerebral cortex, deep gray matter and hippocampus. In comparison, the non-depleted group displayed mild, patchy perineuronal inflammation with occasional deep gray matter and cortical perivascular inflammation. These findings support our hypothesis that CD4^+^ T are important in protecting nervous tissue from damage during ZIKV infection.

As mice age from the weanling stage to adulthood, their adaptive immune systems become more mature, often making them less susceptible to infection. This appears to be the case with mouse models of ZIKV infection, as younger mice tend to suffer much more severe disease burden during ZIKV infection than older mice both in the literature [26] as well as in our hands we saw 100% lethality independent of dose in 4-week-old Ifnar1^-/-^ mice. Therefore, we hypothesized that the consequences of CD4^+^ T cell depletion during ZIKV infection would be difficult to detect in 4-week-old Ifnar1^-/-^ mice. To test this hypothesis we depleted CD4^+^ T cells in 4-week-old Ifnar1^-/-^ mice as described above, and infected with 10^4^ FFU of ZIKV by SC injection (n = 11 control, n = 12 CD4^+^ depleted). Following ZIKV infection we monitored the mice daily, recording: mortality, weight, and clinical signs of disease (S2A and S2B Fig). In a separate experiment, we harvested tissues from these mice at days 4 and 7 post-infection and evaluated viral burden by qPCR (S2C–S2H Fig). The control 4-week-old Ifnar1^-/-^ mice had an earlier onset of mortality and displayed a more dramatic weight loss (S2A and S2B Fig) as compared to the control 10-12-week-old Ifnar1^-/-^ mice (Fig 1), fitting with previous literature. While there were statistically significant differences in mortality of the 4-week-old CD4 depleted vs. control Ifnar1^-/-^ mice (S2A Fig), the CD4 depleted mice did not lose significantly more weight (S2B Fig), and there were no dramatic differences in clinical disease signs (S2C Fig), or viral burden at days 4 or 7 post-infection (S2D–S2I Fig). This result supports our hypothesis, and emphasizes the importance of a *mature* CD4^+^ T cell response for protection from ZIKV. Therefore, we continued our experiments using only Ifnar1^-/-^ mice between the ages of 10–12 weeks old.

### CD4^+^T cells are important for controlling viral titers in the liver and CNS

**Fig 2:**
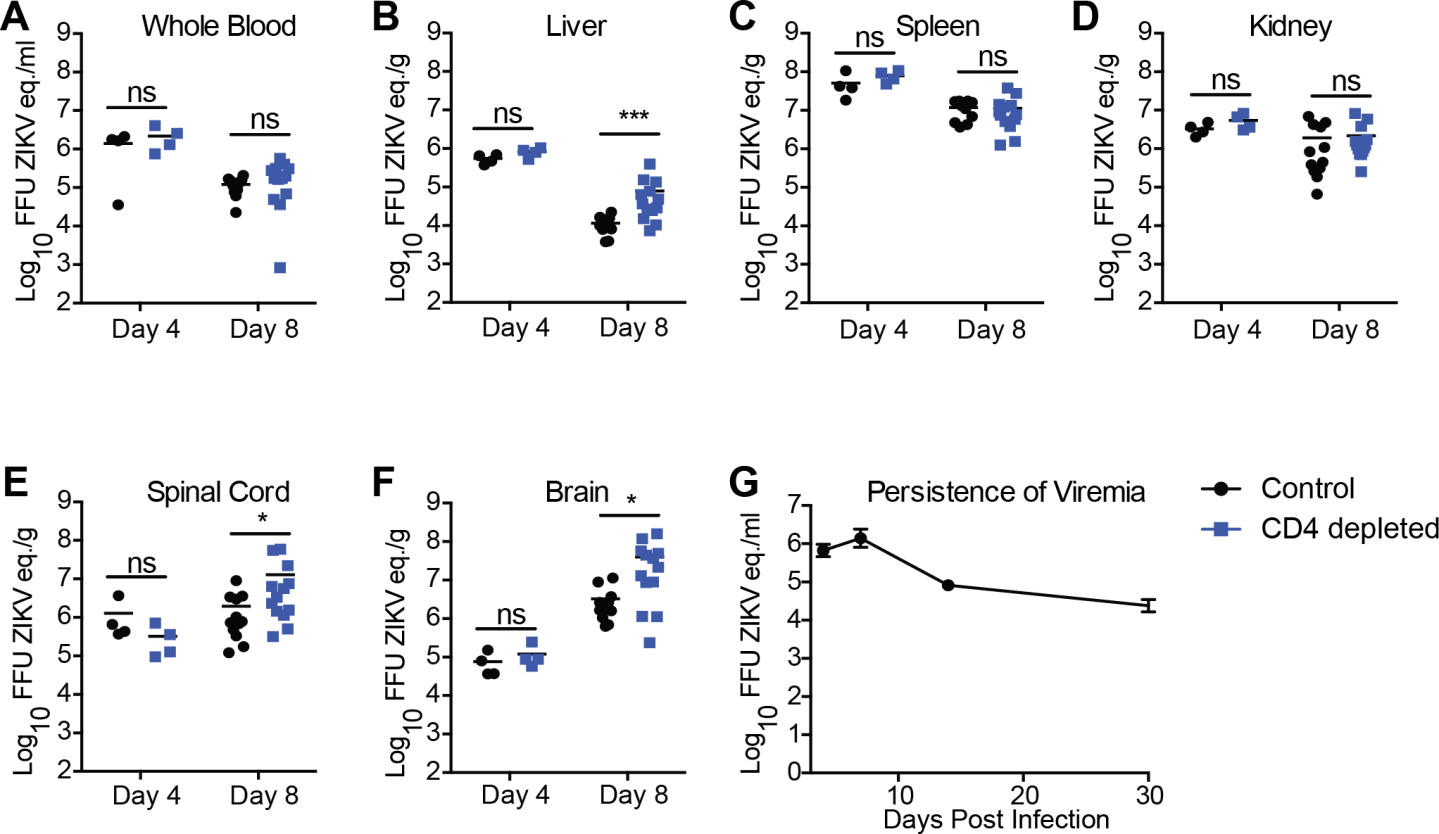
CD4^+^T cells are important for controlling viral replication in the liver and CNS. **A-F.** Viral burden in the peripheral and CNS tissues after CD4^+^ depletion and ZIKV infection. CD4^+^ depleted or control mice were infected with 10^5^ FFU ZIKV via footpad injection. On day 4 (n = 4 per group) or day 8 (n = 12–13 per group) post-infection organs were harvested, snap frozen, weighed, and homogenized. Levels of viral RNA were quantified by qPCR in whole blood **(A)**, liver **(B)**, spleen **(C)**, kidney **(D)**, spinal cord **(E)**, and brain **(F)**. Data are shown as Log_10_ focus-forming unit equivalents (eq.) (as determined by standard curve) per gram or ml of tissue or blood respectively. Data is pooled from 2 independent experiments. Asterisks indicate values that are statistically significant (*, p<0.05, ***, p<0.001) as determined by Mann-Whitney test. **(G)** Viral RNA was measured by qPCR in whole blood of 10-12-week-old mice over 30 days. Over the time monitored, mice were unable to clear the viral RNA.

To determine if CD4^+^T cells were protecting the central nervous system (CNS) from ZIKV infection we measured viral titers in the 10–12 week-old CD4 depleted and control mice at different times post infection. Similar to what was observed by Sitati and Diamond for WNV on day five post infection [35]; we saw no differences in viremia between the CD4 depleted and control mice using qPCR following a sampling of the blood on days four and eight post infection (Fig 2A). We next compared the viral titers in different organs between the CD4 depleted and control mice infected with ZIKV. We compared viral loads in the spleen, liver, kidney, spinal cord and brain by quantitative PCR on days four and eight after ZIKV infection. We noted no differences in the viral titers in the periphery or the CNS (spinal cord or brain) four days after infection by quantitative PCR. Eight days post infection we detected virus in the peripheral organs including liver (Fig 2B), spleen (Fig 2C), and kidneys (Fig 2D) of both the CD4 depleted and control animals. We also detected viral genomes in the spinal cord (Fig 2E) and brain (Fig 2F) of both the CD4 depleted and control mice. We saw significant differences in the ZIKV genome copy number between the depleted and control mice in the liver and significantly higher viral titers in the brains and spinal cords of the CD4 depleted Ifnar1^-/-^ mice as compared to the controls. However, we noted no difference in virus titers in both the spleens and kidneys. Our results are very similar to what has previously been seen for Rift Valley Fever virus [41]; and suggest that the CD4^+^T cell response is playing a role in the protection against the neurological disease associated with ZIKV.

We did not expect to see such high viral titers in both the periphery and the CNS day eight post infection (Fig 2B–2F) in the control Ifnar1^-/-^ mice. This was especially surprising as all of the Ifnar1^-/-^ survive ZIKV infection with this dose and route. To determine if the Ifnar1^-/-^ mice were able to clear ZIKV we looked for ZIKV in long-term survivors. We could detect viremia in the blood by qPCR as late as 30 days post infection (Fig 2G). The persistence of ZIKV in the control Ifnar1^-/-^ mice points to an important role for type I IFN in viral clearance of ZIKV, which we had not previously appreciated. Further studies looking at the implications of long-term persistence of ZIKV on the immune response are currently ongoing.

### CD4^+^T cell depletion did not alter cellular immune responses

**Fig 3:**
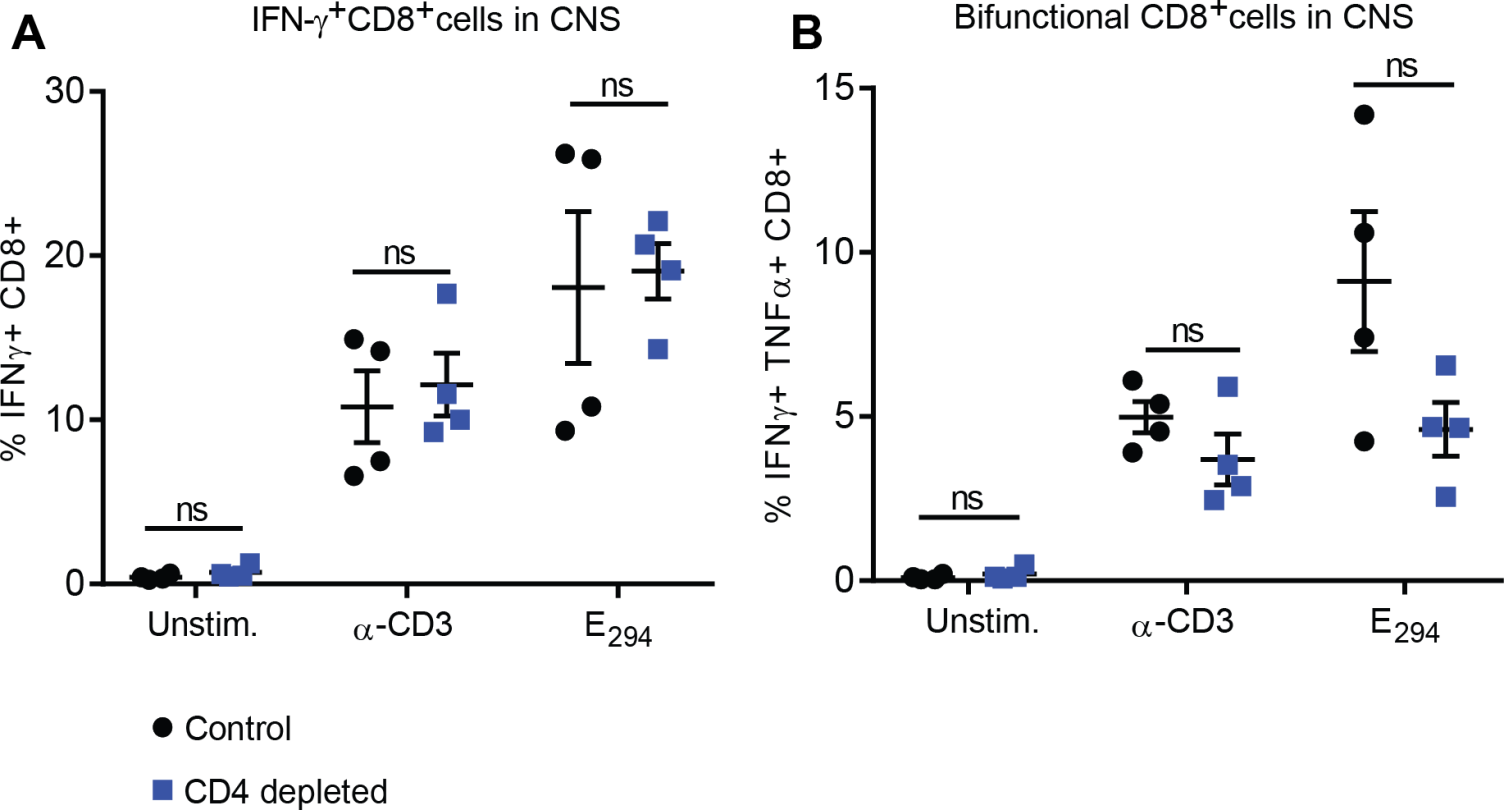
CD4^+^T cell depletion did not alter the CD8^+^ cellular immune response to ZIKV. **(A)** IFNγ production by CD8^+^T cells in response to stimulation with anti-CD3 or CD8^+^ epitope E_294_. On day 8 post-infection, ten- to twelve-week-old depleted or control mice (n = 4 per group) were perfused with 20 ml of PBS. The brains were harvested and lymphocytes were purified as described in the methods section. Lymphocytes were stimulated with CD8^+^ peptide epitope E_294_ or anti-CD3 in the presence of brefeldin A, stained and analyzed by flow cytometry. Cells were gated on a lymphocyte gate, CD3^+^, CD19^-^, CD4^-^, CD8^+^ and were analyzed for functional response by expression of IFNγ. The differences between the control and depleted groups in IFNγ production by CD8^+^T cells were not statistically significant (p>0.66) as evaluated by Mann-Whitney test. **(B)** To assess the overall quality of the CD8^+^T cell response to these stimuli, cells were also analyzed by flow cytometry for IFNγ and TNFα bifunctionality in response to stimulation in the presence of brefeldin A. The differences between the control and depleted groups in CD8^+^T cell bifunctionality were not statistically significant (p>0.2) as evaluated by Mann-Whitney test. Data is from a single experiment (n = 4).

Previous studies have shown the importance of CD8^+^T cells in controlling ZIKV infection [24, 29, 30, 40]. Based on these previous studies and our observations that the CD4 depleted mice had signs of a CNS disease, we hypothesized that the absence of CD4^+^T cells would lead to an increase in CD8^+^T cell mediated inflammation in the brains of ZIKV infected mice. To test this hypothesis, we examined the CD8^+^T cell response in the brains of depleted mice to determine if the absence of CD4^+^T cells altered the CD8^+^T cell response to ZIKV. As described above we depleted CD4^+^T cells from Ifnar1^-/-^ mice prior to infection with ZIKV. On day eight post ZIKV infection we harvested the brains of four CD4 depleted and control mice. Looking at the total IFN-γ producing ZIKV specific CD8^+^T cells responding to CD3 stimulation we saw an equal percentage of CD8^+^T cells in the brains of the depleted and control mice (Fig 3), and the amount of IFN-γ produced on a per cell basis (IFN-γ geometric mean fluorescent intensity) between the two populations was not significantly different. Similarly, when we examined the IFN-γ response to the immunodominant CD8^+^T cell epitope E_294_ we saw no differences in the responding CD8^+^T cells in the brains of the depleted and control mice. This result suggests that the absence of CD4^+^T cells did not alter the CD8^+^T cell response to ZIKV in the brains of our animal model.

To evaluate the cellular inflammatory response during infection we also evaluated clinical hematology from the CD4 depleted and control animals on days four and eight post infection (Table 1). On day four post infection we did not see any differences in the depleted versus control animals. Neutrophils, lymphocytes and monocytes in the CD4 depleted and control infected mice were slightly higher than the range determined for uninfected aged match C57BL/6J mice (JAX phenome). On day eight post infection the CD4 depleted mice showed a significant difference in the number of neutrophils present compared to controls although there was no significant difference in the other hematological parameters measured suggesting that the CD4 depletion did not dramatically alter inflammatory cell populations.

**Table 1:** Hematology on CD4^+^T cell depleted or control Ifnar1-/- mice following ZIKV infection. Ten- to twelve-week-old Ifnar1-/- mice were depleted and infected as described in Fig 1. Mice were bled into EDTA tubes on days four and eight post infection and hematological parameters were assessed using an IDEXX ProCyte Dx hematology machine.

	Day 4 Ifnar1^-/-^		Day 4 Ifnar1^-/-^ Depleted		
	Average K/μl	STDV	Average K/μl	STDV	p =
White Blood Cells #	3.00	1.37	3.13	.039	0.83
Neutrophils #	1.25	0.59	1.71	0.49	0.26
Lymphocytes #	0.96	0.57	0.78	0.20	0.45
Monocytes #	0.73	0.55	0.57	0.23	0.86
Eosinophils #	0.06	0.04	0.06	0.01	0.85
Basophils #	0.01	0.01	0.01	0.01	0.36
	Day 8 Ifnar1-/-		Day 8 Ifnar1-/- Depleted		
	Average K/μl	STDV	Average K/μl	STDV	p =
White Blood Cells #	9.06	2.00	12.00	2.71	0.09
Neutrophils #	3.99	1.50	7.22	1.89	0.02
Lymphocytes #	2.96	2.04	2.45	1.47	0.66
Monocytes #	1.16	0.70	2.01	1.22	0.21
Eosinophils #	0.12	0.12	0.31	0.43	0.36
Basophils #	0.01	0.01	0.01	0.01	0.77

### ZIKV specific CD4^+^T cell epitopes identified

To identify mechanisms of CD4^+^T cell-mediated protection we determined both the breadth and magnitude of the antigen specific CD4^+^T cell response. First, antigen specific CD4^+^T cells populations were identified utilizing a ZIKV-specific peptide library. ZIKV-specific amino acid sequences were used to generate a library of fifteen-mer peptides overlapping by ten amino acids. The library generated was derived using the published sequence for ZIKV PRVABC59 (Accession number KU501215.1) [42], resulting in 683 individual peptides (S1 Table) spanning the entire polyprotein.

To identify CD4^+^T cell epitopes in this library, we first infected C57BL/6J mice with 1x10^6^ FFU of ZIKV intravenously (IV). After 30 days, the mice were boosted with 1x10^6^ FFU of ZIKV IV and sacrificed four days after the boost. The splenocytes isolated from the ZIKV boosted animals were plated into 96 well plates and stimulated in the presence of Brefeldin A (BFA) for six hours with pools of six to eight peptides spanning different regions of the genome. Anti-CD3 (45-2C11) was used as a positive control, to stimulate antigen experienced CD4^+^T cells, and a well of unstimulated cells was setup as a negative control. After stimulation, the splenocytes were stained with the cell surface antibodies directed against, CD3, CD8, CD4 and CD19. The cells were then fixed, permeabilized and stained intracellularly with antibodies specific for the mouse cytokines Interferon-γ (IFN-γ) and tumor necrosis factor-α (TNF-α). Splenocytes were then run on an LSRII and CD4^+^T cells were analyzed for cytokine production in response to stimulation from the peptide pools. The cytokine responses detected from the pooled peptide wells allowed us to identify 6–36 possible CD4 epitopes. To identify the individual peptides within the peptide pools the infections and boost were repeated in four-month-old C57BL/6J. The peptide pools were split out and the individual peptides that respond to ZIKV were identified (Table 2). Through these studies, we identified 18 epitopes where the amount of IFN-γ produced in response to peptide stimulation was higher than our background control unstimulated cells in at least one mouse during our prime boost experiments.

**Table 2:** Identified ZIKV-specific CD4^+^T cell epitopes. Utilizing a full length ZIKV peptide library, 18 ZIKV-specific CD4^+^ T cell epitopes were identified by ex vivo restimulation of splenocytes from ZIKV infected C57BL/6J mice (H2-b). Once epitopes were identified, they were assigned amino acid residue numbers based on the location of their start site within the polyprotein. Shaded in grey are the four immunodominant epitopes that were present both in the acute and memory responses to ZIKV.

Plate Position	ZIKV protein	Amino Acid sequence
10	C_46_	[H]MVLAILAFLRFTAIK[OH]
14	C_66_	[H]INRWGSVGKKEAMET[OH]
51	PrM_251_	[H]IFRNPGFALAAAAIA[OH]
55	PrM_271_	[H]STSQKVIYLVMILLI[OH]
119	E_591_	[H]KGVSYSLCTAAFTFT[OH]
130	E_646_	[H]GRLITANPVITESTE[OH]
163	NS1_811_	[H]TGVFVYNDVEAWRDR[OH]
282	NS2b_1406_	[H]AVGLLIVSYVVSGKS[OH]
298	NS2b_1486_	[H]IPFAAGAWYVYVKTG[OH]
302	NS3_1506_	[H]LWDVPAPKEVKKGET[OH]
345	NS3_1721_	[H]TVILAPTRVVAAEME[OH]
407	NS3_2031_	[H]RKTFVELMKRGDLPV[OH]
434	NS4a_2166_	[H]QLPETLETIMLLGLL[OH]
437	NS4a_2181_	[H]GTVSLGIFFVLMRNK[OH]
474	NS4b_2366_	[H]MIGCYSQLTPLTLIV[OH]
486	NS4b_2426_	[H]IDTMTIDPQVEKKMG[OH]
610	NS5_3046_	[H]IPGGRMYADDTAGWD[OH]
643	NS5_3211_	[H]KDTQEWKPSTGWDNW[OH]

**Fig 4:**
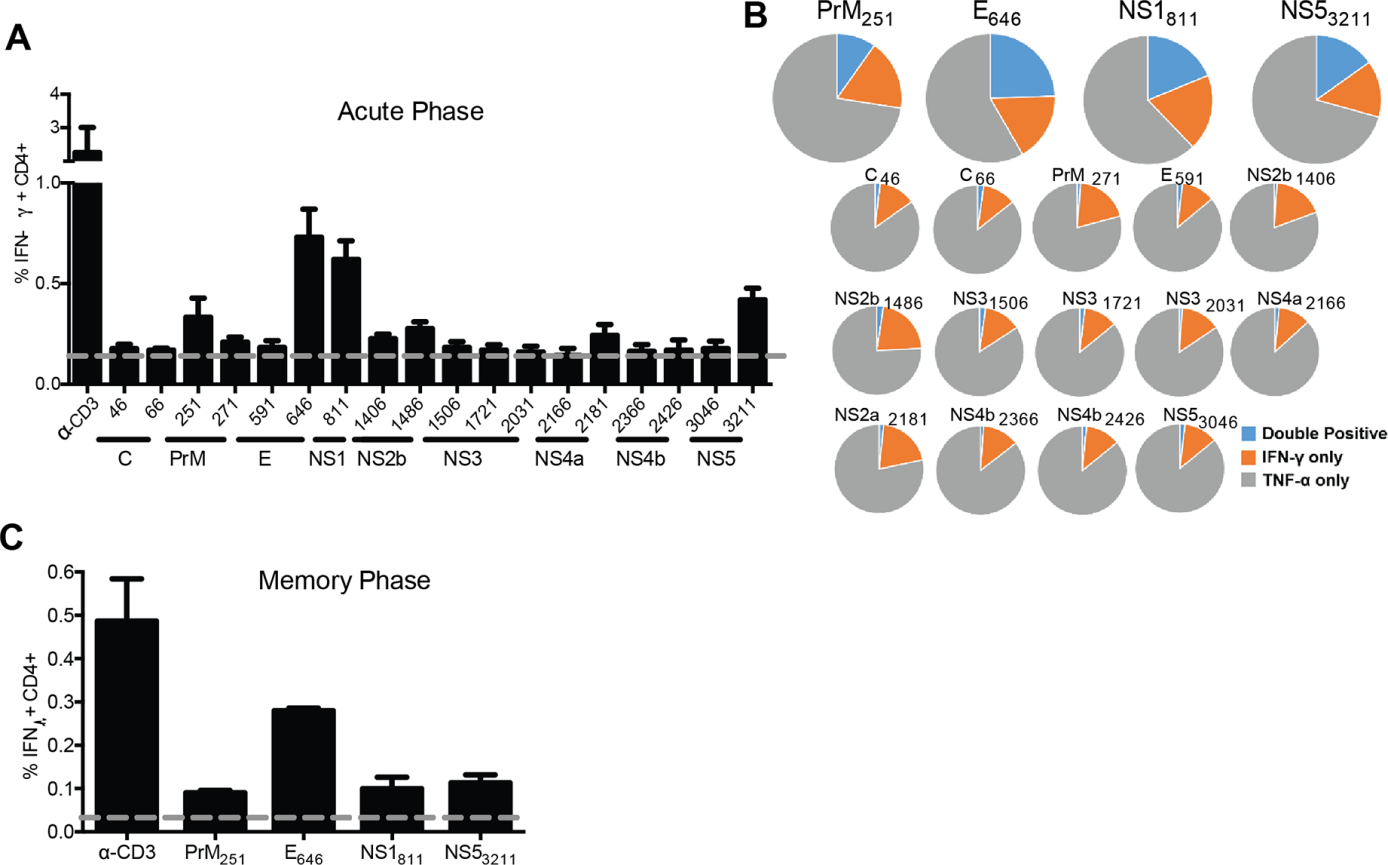
Identification and functional analysis of ZIKV-specific CD4^+^T cell epitopes. **(A)** ZIKV-specific CD4^+^T cell epitope identification in the acute phase of infection. C57BL/6J mice (n = 3) were injected IV with 10^5^ FFU of ZIKV. At 10 DPI splenocytes were harvested and stimulated with 10 μg of the indicated peptide in the presence of brefeldin A. Cells were stained for surface markers (CD3, CD19, CD4 and CD8), stained intracellularly for IFNγ and TNFα and analyzed by flow cytometry. Cells were gated using a lymphocyte gate, CD19^-^, CD4^+^, CD8^-^ and were functionally analyzed by expression of IFNγ. Data is presented as the percent of CD4^+^T cells that produced IFNγ in response to stimulation. The dashed line represents the background level of IFNγ production by unstimulated cells. Peptides were classified as hits if the percent of CD4^+^T cells producing IFNγ was two-fold over background in at least 1 mouse. 18 epitopes were identified, including 4 dominant epitopes. **(B)** Bifunctionality of response to each epitope. The quality of the response to each epitope during acute infection (n = 3) was evaluated and is represented in the pie charts as the percentage of the total antigen specific cells (IFNγ and/or TNFα positive) which are either positive (gold or grey) or double positive (blue). **(C)** Functional responses to the immunodominant epitopes are preserved in memory. More than 30 DPI, splenocytes were harvested from infected C57BL/6J mice (n = 3) and were stimulated and stained as described above. Data is presented as the percent of CD4^+^T cells that produced IFNγ in response to stimulation. The dashed line represents the background level of IFNγ production by unstimulated cells. Data is from a single experiment (n-3).

We next set out to determine the expression hierarchy of the identified peptide epitopes during the acute infection. Similar to the epitope identification experiments, C57BL/6J mice were infected IV with 1x10^6^ FFU of ZIKV and ten days after infection the mice were sacrificed and splenocytes were isolated. The splenocytes were stimulated with the identified individual peptides in the presence of BFA then stained for cell surface and intracellular cytokines to identify the antigen specific cells (Fig 4A). Using this technique, we confirmed all of the 18 CD4^+^T cell epitopes that we identified from our screen. Each of the epitopes was detected above background in at least one animal. We named the ZIKV peptide epitopes using the same nomenclature as used previously for WNV [43], with the abbreviated name of the viral protein followed by the number of the amino acid based upon the flavivirus open reading frame, for example E_646_. We then looked at the proportion of the acute CD4^+^T cell response dedicated to each of these ZIKV specific CD4^+^T cell epitopes and if the responding epitope specific population was making IFN-γ, TNF-α or both cytokines (Fig 4B). We noted that the CD4^+^T cells that recognize epitopes with a higher frequency (immunodominant epitopes), as represented by the larger circles (E_646_, prM_251_, NS1_811_, and NS5_3211_), were also the most polyfunctional having the greatest portion of the responding cells making IFN-γ and TNF-α following peptide stimulation. Of note, this method was also used to evaluate ZIKV-specific CD8^+^ T cell responses and was able to confirm a robust response to the immunodominant E_294_ D^b^ restricted epitope as previously reported [44]

To determine if the epitope specific CD4^+^T cell response was preserved into memory we next repeated our infection of C57BL/6J mice with an IV injection with 1x10^6^ FFU of ZIKV and waited 30 days before harvesting the spleens and performing the intracellular cytokine stain (Fig 4C). While 18 epitopes were detectable during the acute infection, only the four immunodominant epitopes were detectable in all mice during the memory infection (prM_251_, E_646_, NS1_811_, and NS5_3211_). Of these four peptides, the response of the E_646_ was consistently dominant in the response and comprised between 0.2–0.4 percent of the total CD4^+^T cell population. The detection of these four CD4 epitopes into memory suggests that the ZIKV specific CD4^+^T cell response is preserved.

Worth noting, the immunodominant CD4^+^ T cell epitopes identified (PrM_251_, E_646_, NS1_811_, and NS5_3211_) appear to be quite conserved across different strains of ZIKV. We compared amino acid residues at 15-mer loci PrM_251_, E_646_, NS1_811_, and NS5_3211_ from 6 different strains of ZIKV to that of the reference library (PRVABC59) (S2 Table). Three strains of Asian lineage were compared including R103451 (GenBank:KX694534), P6-740 (GenBank:KX377336), and FLR (GenBank: KU820897). Three strains of African lineage were also compared to the reference library including MR766 (GenBank:KX377335), DAK AR (GenBank:KY348860), and IbH (GenBank:KU963574). Epitopes PrM_251_, E_646_ and NS1_811_ were 100% conserved across all Asian lineages of ZIKV assessed, while epitope NS5_3211_ was 98.3% conserved across the Asian lineages. Epitope PrM_251_ was 86.7% conserved across the African lineages assessed. Epitope E_646_ was 100% conserved across the African lineages. Epitope NS1_811_ was 96% conserved across the African lineages and epitope NS5_3211_ 93.3% conserved across the African lineages.

### Analysis of CD4^+^T cell TCRβ diversity

**Fig 5:**
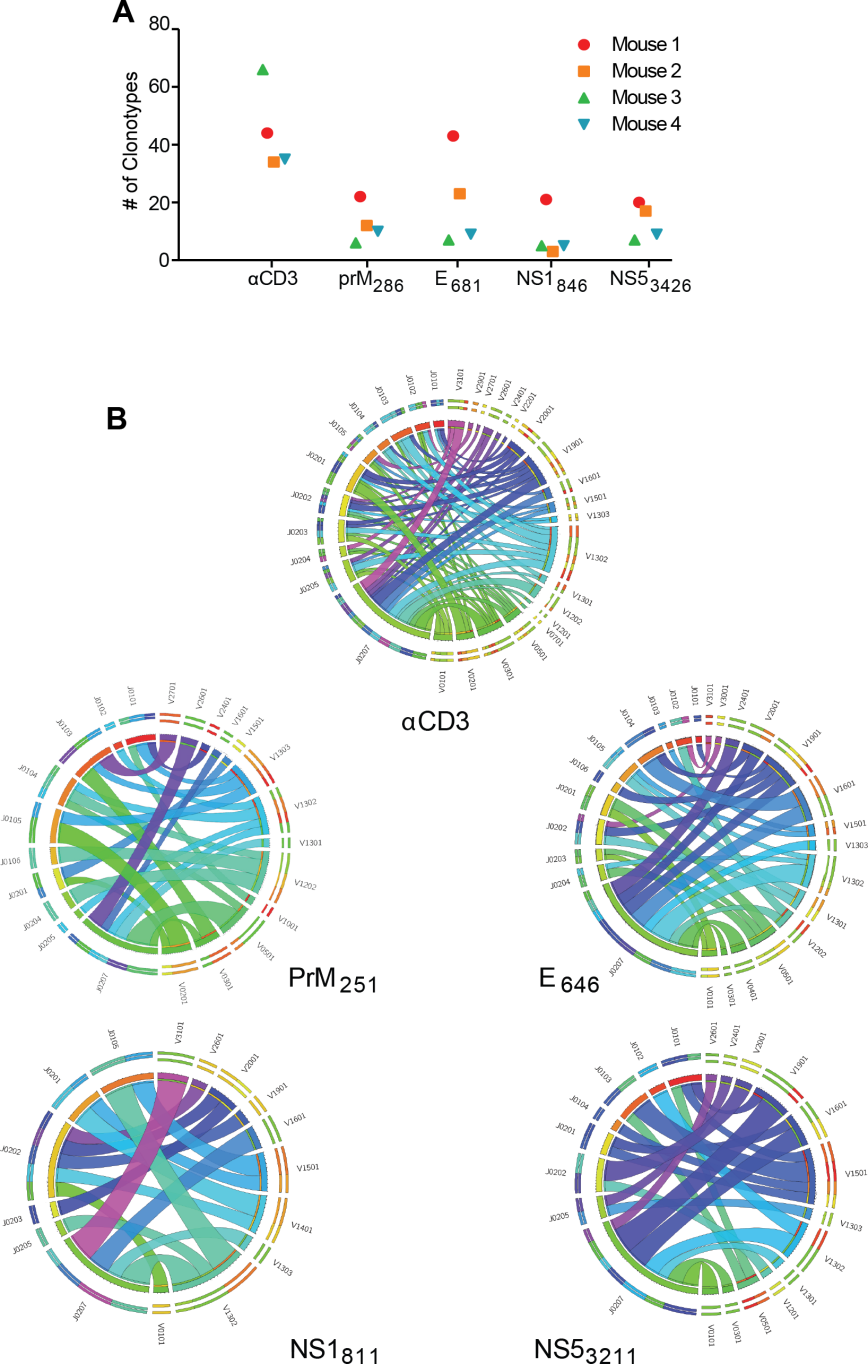
ZIKV-specific CD4^+^T cell TCR diversity. **(A)** TCRβ CDR3 region diversity among CD4^+^T cells responding to ZIKV-specific epitopes. Wild type C57BL/6J mice (n = 4) were infected with 10^6^ FFU ZIKV. At 10 DPI, splenocytes were harvested and stimulated with the indicated peptide or anti-CD3 in the presence of brefeldin A as described in Fig 4. CD19^-^, CD8^-^, CD4^+^, IFNγ^+^ cells were sorted separately for each peptide and DNA was extracted for multiplexed amplification of the TCRβ CDR3 region. Unique CDR3 sequences (clonotypes) are enumerated for cells responding to each peptide for each mouse. Each line represents the number of unique TCRβ clonotypes identified from individual peptide or αCD3 stimulated T cells from four mice. **(B)** Representation of unique TCRβV-J recombinations in cells responding to each stimulation. Circos graphs were generated to display the relative frequency of each specific TCRβV-J join detected in CD4^+^T cells responding to each epitope. The width of each ribbon is proportional to the number of unique clonotypes that share those specific TCRβV-J segments.

Having identified several CD4^+^T cell epitopes that generate polyfunctional responses to ZIKV, we next examined the clonality of the TCRs expressed by ZIKV specific CD4^+^T cells (oligoclonal vs. polyclonal). We determined the TCR V-β sequences within the antigen specific CD4^+^T cells isolated during acute ZIKV infection (Fig 5A and 5B). Four C57BL/6J mice were infected with 10^6^ FFU ZIKV and splenocytes isolated ten days post infection. Splenocytes from each of the four mice were separated into 5 pools and stimulated in the presence of BFA with the identified CD4^+^T cells epitopes prM_251_, E_646_, NS1_811_ and NS5_3211_, or anti-CD3 for six hours as done previously for the epitope identification. Samples were then stained with cell surface and intracellular antibodies for CD8, CD4, CD19, and IFN-γ. Cells from the peptide or CD3 stimulated samples were then sorted on CD8^-^CD19^-^CD4^+^IFN-γ^+^to isolate the epitope specific T cells. DNA was isolated, TCRβ sequences were amplified, and TCRβV-D-J gene usage was determined by next generation sequencing to identify TCRβ sequences associated with each ZIKV CD4^+^T cell-epitope.

To assess the diversity of the response, we determined the number of unique TCRβ sequences expressed in the cells producing IFN-γ in response to each individual ZIKV epitope. On average, we identified 6–22 unique TCRβ sequences per mouse that recognized prM_251_, 7–43 TCRβ sequences to E_646_, 3–21 to TCRβ sequences NS1_811_ and 9–20 TCRβ sequences to NS5_3211_ (Fig 5A). Further analyses revealed a diverse array of different TCRβ chains expressed by CD4^+^T cells responding to each ZIKV peptide (Fig 5B). The Circos graphs [45] represent the sum of all unique TCRβV-TCRβJ recombinations in TCR repertoires of sorted CD4^+^IFN-γ^+^ T-cells after stimulation with αCD3 or ZIKV-specific peptides. The width of the ribbon is representative of the number of unique clonotypes within the repertoire that share particular V-J segments. Of note, each mouse generated a “private” T cell response to ZIKV in that the same receptors were not identified in different mice. Together these data demonstrate that the CD4^+^T cell response to ZIKV is comprised of a diverse array of TCR sequences to each peptide (polyclonal), and largely unique to each individual.

### CD4^+^T cells are sufficient to protect against lethal ZIKV challenge

**Fig 6:**
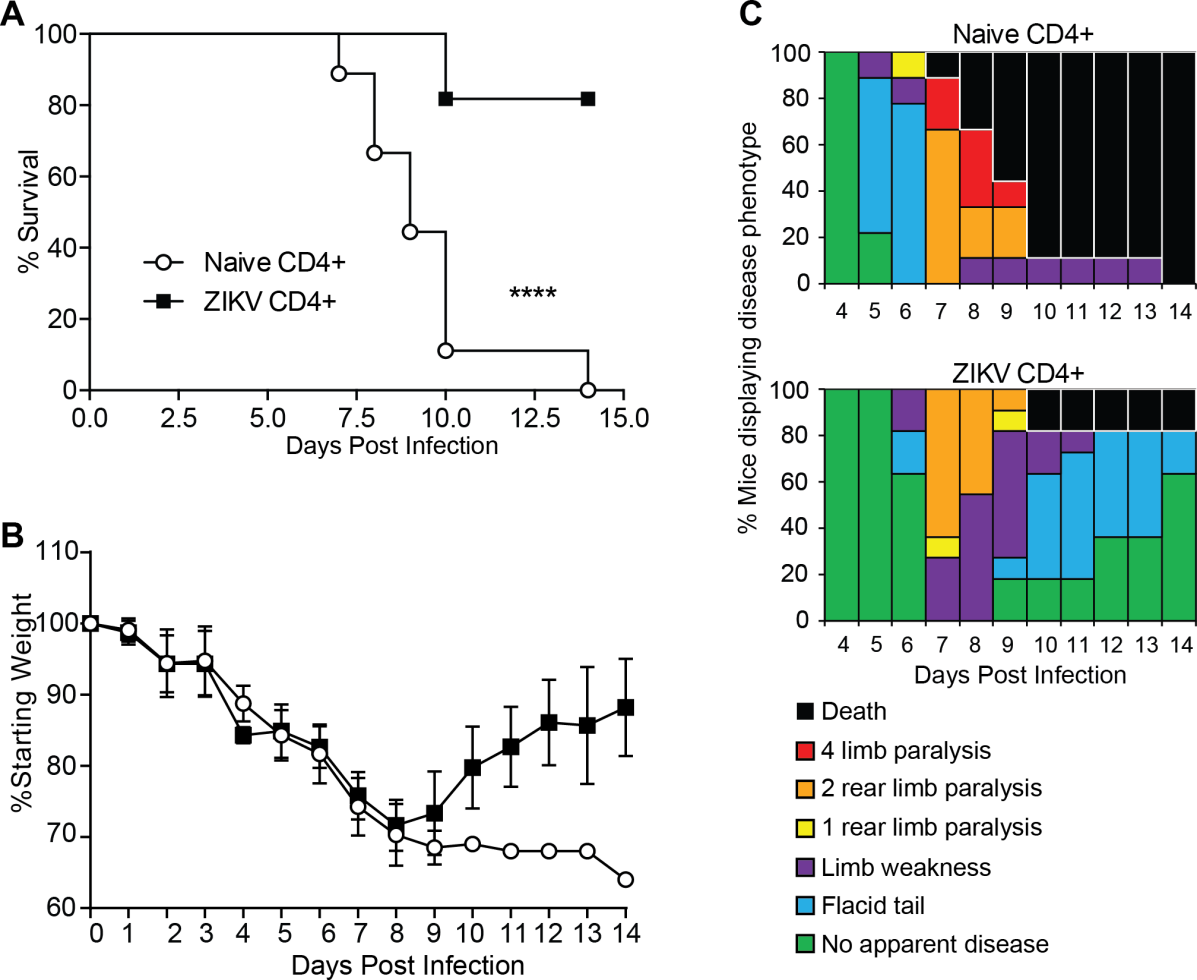
CD4^+^T cells are sufficient to protect against a lethal ZIKV challenge. **(A)** Survival of ten- to twelve-week-old mice following adoptive transfer of CD4^+^T cells and IV route ZIKV challenge. At 30 days post infection, CD4^+^T cells were isolated to >97% purity from ZIKV infected or naïve C57BL/6J mice and transferred IV into ten- to twelve-week-old Ifnar1^-/-^ mice (~3x10^6^ /mouse) 1 day prior to IV infection with 10^5^ FFU of ZIKV (n = 9–11 per group). Survival differences were statistically significant between the two groups (p<0.0001) as determined by Mantel-Cox test. **(B)** Weight loss during IV ZIKV infection of ten- to twelve-week-old mice following adoptive transfer. As a measure of disease, mice were weighed daily for 14 days. No statistically significant differences in weight loss were observed. **(C)** Neurological sequela associated with IV ZIKV challenge following adoptive transfer. Mice were evaluated for signs of neurological disease daily and graphed on each day as a percentage of mice displaying that disease indicator. Signs of disease range and in the most severe cases accelerate in the following manner from no apparent disease, limp tail, hind limb weakness, hind limb paralysis, complete paralysis and death. Data is pooled from 2 independent experiments.

Finally, we sought to determine if virus specific CD4^+^T cells were sufficient for protection. We adoptively transferred CD4^+^T cells isolated from either naïve or ZIKV infected C57BL/6J mice Ly5.1. C57BL/6J mice were infected with ZIKV and 30 days later splenocytes were harvested from either infected or naïve mice and CD4^+^T cells isolated by negative selection using magnetic beads with a purity of approximately 96 percent. We then adoptively transferred 4 x 10^6^ CD4^+^T cells from either group into 10-12-week-old Ifnar1^-/-^ mice one day prior to lethal challenge with ZIKV (intravenous, 10^5^ FFU). We monitored the morbidity, mortality and signs of disease of the ZIKV infected Ifnar1^-/-^ mice for 14 days (Fig 6A–6C). While the majority of Ifnar1^-/-^ mice that received the CD4^+^T cells from the ZIKV immunized mouse survived, 100% of the mice that received the naive CD4^+^T cells succumb to the lethal challenge (Fig 6A). Due to high mortality in the control group, there were not enough n to statistically evaluate differences in weight loss, though mice that received CD4^+^ T cells from ZIKV immunized mice began to recover starting at day 9, the 1 remaining mouse that received naïve CD4+ T cells continued to deteriorate until death on day 14 (Fig 6B) and while most mice developed hind limb paralysis mice that received CD4^+^T cells from ZIKV immunized mice were able to recover (Fig 6C). While the CD4^+^T cell depletion experiments demonstrate that the CD4^+^T cells are necessary for protection, these results indicate that ZIKV specific CD4^+^T cells are sufficient to protect against lethal ZIKV infection.

## Discussion

As we begin to develop a deeper understanding of the correlates of protection against flavivirus disease, we are appreciating that the combination of both the adaptive cellular and humoral responses are required for protection against many flaviviral pathogens. The generation of a strongly neutralizing antibody response has long been the gold standard for immune mechanism that correlate with protection for many infections; however, the role of T cell responses in vaccine mediated protection against viral disease has become clear. For example, the strong and broad T cell response generated by the yellow fever vaccine (YFV-17D) (reviewed in [46]) is thought to contribute to its high efficacy. As with YFV, recent studies have suggested that strong T cell responses may also be important for controlling DENV infections and for reducing the possible effects of antibody dependent enhancement (ADE) [47, 48]. While our understanding of the cellular immune responses that develops during the natural course of flavivirus infection has begun to contribute to our understanding of flavivirus correlates of protection, there are still significant gaps in our knowledge of how to generate a protective immune response against this family of emerging pathogens.

Here we have identified a role for CD4^+^T cells in protection from ZIKV-induced neurologic disease, and viral control using an established mouse model. Through depletion studies we demonstrated that CD4^+^T cells are necessary for the protection against lethal ZIKV challenge (Fig 1B). We noted a significant difference in weight loss (Fig 1C) and a marked difference in the signs of neurological disease between the CD4 depleted and control (Fig 1D) indicating that CD4^+^T cells are important for protection of the Ifnar1^-/-^ mice from a CNS associated disease. On day four post infection viral loads in the tissues of the depleted and control mice were not significantly different, suggesting the innate immune response was not impacted by the absence of CD4^+^T cells in our animal model. By 8 days post infection CD4-depleted mice develop higher viral RNA (vRNA) titers in liver, spinal cord and brain than control mice, indicating that CD4^+^T cells were necessary to control virus replication in the liver and CNS (Fig 2). Importantly, higher levels vRNA in the CNS of the depleted mice was associated with increased signs of neurological disease including whole body tremors and a significantly greater incidence of limb paralysis. The non-depleted mice did not manifest as severe neurologic disease as the CD4 depleted mice, although we were able to detect vRNA in the brains of these mice. We therefore propose that ZIKV invasion of the CNS can occur independent of CD4^+^T cells however CD4^+^T cells are important to control viral replication and prevent the exacerbation of severe neurological disease.

Studies investigating the T cell response for ZIKV in both humans and mice have identified a role for CD4^+^T cells in responding to ZIKV infection ([24, 29, 49]) although their importance and sufficiency in the protection against ZIKV morbidity and mortality has not been clearly defined. While several studies have shown the CD4^+^T cells responding to ZIKV infection can produce cytokines [24, 49] other studies point to a more dominant role of CD8^+^T cells in controlling ZIKV infection [24, 40]. Our studies define a protective role for CD4^+^T cells in controlling virus in the livers and CNS (Fig 2B–2F) as well as preventing lethality (Fig 6). Supporting the role of CD4^+^T cells in preventing a CNS disease, we saw that in the absence of CD4^+^T cells mice infected with ZIKV had severe body tremors and rapid paralysis. Histological analysis of the brains of the CD4-depleted and control groups noted inflammation in brains of all infected animals but we saw an elevated severity of inflammation associated with the CD4 depleted animals (S1 Fig). While this is interesting, more studies would need to be done to determine this exact mechanism.

We did also note that hind limb paralysis was not an accurate surrogate for death from ZIKV infection, as all the mice in the control group and twenty percent of the mice in the CD4-depleted group recovered within 48 hours of first noting paralysis with no evidence of disease by 72 hours (Fig 1D). We noted a similar discrepancy in our CD4 adoptive transfer experiment (Fig 6), where clinical score and weight loss were not accurate surrogates of survival. The challenge going forward is to provide a clear picture of the correlates of protection against ZIKV disease in animal models the context of studies using variable surrogate disease markers as indicators of mortality.

The impact of CD4^+^T cells on the adaptive immune response is multifaceted extending to development of the humoral immunity, direct cytolytic activity, production of cytokines and chemokines for cell recruitment, providing CD8^+^T cell help and regulation of the immune response. The outcome of the CD4^+^T cell specific recognition of the pathogen is dictated by the many factors including the environment in which the recognition occurs, and the previous experience of the CD4^+^T cell with the specific invading pathogen. In the case of flaviviruses, for both DENV and WNV, the inclusion of CD4^+^T cell epitopes in the experimental vaccines have increased the immunogenicity of the vaccines [50]. The identification of the CD4^+^T cell epitopes within animal models of ZIKV disease would then allow for further investigation into correlates of protection as well as possible avenues for improving vaccine efficacy.

With the alterations in disease we observed after CD4^+^T cell depletion and the role of CD4^+^T cells in vaccine efficacy for WNV and DENV, we set out to identify H2-IA^b^-restricted epitopes, the first ZIKV specific CD4^+^T cell epitopes defined in mice. By means of an overlapping peptide library and functional identification using intracellular cytokine responses, we were able to identify MHCII-restricted CD4^+^T cell epitopes, corresponding to the prM protein (prM_251_), Envelope (E_646_) and the nonstructural proteins NS1 (NS1_811_) and NS5 (NS5_3211_). Using these newly identified MHCII-restricted epitopes we defined a robust CD4^+^T cell response during the acute phase that persisted into the memory phase.

The CD4^+^T cell epitope E_646_ is found within domain III of the ZIKV envelope. This is significant as many of the strongly neutralizing antibodies for flaviviruses including ZIKV are directed to this domain [51–54]. As we move forward with a rationale vaccine design strategy, one important factor is the inclusion of CD4 epitopes within protein domains that contain targets for strongly neutralizing antibodies [55]. CD4 T cell help and the cytokine environment drive affinity maturation of the B cell population by linked recognition [55]. We hypothesize that as vaccines designed to elicit strong neutralizing antibody response to ZIKV are tested, the inclusion of E_646_ in the vaccine construct may help to generate protective antibody responses in mouse models, and illustrate the importance of defining T cell epitopes for the human population. Additionally, broader analyses suggest that CD4^+^ responses in ZIKV infected humans are often targeted to both structural and non-structural elements of the virus, underscoring the potential relevance of our findings in vaccine design [56, 57].

It should be noted that we used immune competent C57BL/6J mice for our ZIKV epitope identification studies. Early on in our studies we had attempted to identify the CD4^+^T cell epitopes in Ifnar1^-/-^ mice infected with ZIKV. We surmised that as the Ifnar1^-/-^ mice were more sensitive to ZIKV infection than the immune competent C57BL/6J, and would therefore have higher antigen loads in the Ifnar1^-/-^ mice resulting in stronger ZIKV-specific CD4^+^T cell responses. However, in our initial screening assays we did not detect strong ZIKV-specific CD4 or CD8 T cell responses in the Ifnar1^-/-^ mice compared to the immune competent C57BL/6J. We also noted that the more susceptible Ifnar1^-/-^ mice that did not succumb to infection had viral titers persist for greater than 30 days after infection with ZIKV, possibly leading to a more exhausted T cell population. As we relied on a functional assay with cytokine production to map the T cell response in our animals we were not able to further investigate this observation. We are currently conducting further studies using CD4 and CD8 T cell tetramers to investigate this observation.

Our development of a whole genome peptide library allowed us to use an unbiased approach to identify the CD4^+^T cell epitopes. We noted that the epitopes identified spanned the genome with dominant epitopes present in both structure (prM_251_ E_646_, an) as well as non-structural (NS1_811_ and NS5_3211_) proteins. However, a limitation of our studies is the use if IFNy and TNFα as a means to identify ZIKV-specific CD4^+^T cell epitopes. Using this approach, we biased our results toward Th1 cells. Further studies to identify ZIKV specific CD4^+^T cell responses other than Th1 responses identified here will need to be carried out to truly gain a more accurate picture of the total CD4^+^T cell response to ZIKV.

On the opposite side of the antigen specific immune response is the CD4^+^T cell and more specifically the CD4^+^T cell receptor. The peripheral TCR repertoire is dependent on the generation of appropriate TCRα/β chains upon somatic recombination of V(D)J gene segments during T-cell development therefore the virus specific TCRs are present prior to exposure to the specific pathogen. The diversity of the TCR response can vary with antigen specificity, meaning that certain epitope specific responses have a highly diverse responding TCR repertoire while other epitope specific TCR responses are more constrained [58]. This variation can be related to both the peptide MHC interactions with the TCR as well as genetic constraints in the generation of the diverse TCR repertoire [58]. Whether the epitope specific CD4^+^T cell response utilizes a broad or narrow set of TCRs does have implications for the outcomes to infections. Previous studies have shown that protection and clearance from viral infection is related to TCR diversity [59–62] with limited TCR diversity being linked to poor disease outcome [60]. The reasons for the improved efficacy of the T cell response being linked to a high level of diversity remains an active area of investigation [58]. Our TCRβ sequencing results (Fig 5) are in agreement with studies that reported a high degree of sequence diversity in the virus specific TCRβ [58, 60, 63, 64] and highlight the need for further experiments to study the implications of this diversity in the mounting of a protective immune response to ZIKV infection.

In our study after identifying the CD4 specific T cell epitopes we shifted our focus toward identifying the level of TCR repertoire diversity. Knowing that CD4^+^T cells were required for control of ZIKV infection and that we could detect a strong antigen specific CD4^+^T cell response in the mice, we hypothesized that the TCR repertoire specific for the different CD4 epitopes would contain a high degree of diversity. Using techniques we have previously established to identify TCRβ sequences diversity of CD4^+^T cells [65], we noted a striking degree of diversity in the TCRβ sequences for an individual ZIKV-specific epitope within a mouse and between mice (Fig 5). We were unable to detect shared “public” TCRs for any epitope between mice confirming our hypothesis that the ZIKV epitope specific T cell responses were clonally diverse suggesting a broadly reactive T cell pool. It should also be noted that many previous studies evaluating the importance and implications of TCR diversity on the immune response were completed with CD8^+^T cells, leaving open the question of the impact of TCR diversity in CD4^+^T cells in controlling ZIKV infection. In our study, we examined the diversity of the ZIKV epitope-specific CD4^+^T cells further studies to understand the functional implications of CD4 TCR diversity as well as the identification of ZIKV-specific public TCRs are ongoing.

For ZIKV we have shown that there is a broad T cell response generated against the pathogen. We have shown that the CD4 cell response is required for protection against a lethal ZIKV challenge. A strength of these studies is the stringency of the animal model for protection that we are using. Many current studies use clinical score including twenty percent weight loss or hind limb paralysis as surrogate markers for mortality. However, in cooperation and under careful observation by our veterinary staff we have demonstrated that mice can completely recover from hind limb paralysis. Importantly these surrogate markers may have masked the effects of some immunological responses. As we move forward to analyze both the correlates of protection as well as establishing small animal models to test the efficacy of vaccines and therapeutics we need to remain mindful that the surrogate morbidity markers currently used to define mortality may mask some protective or detrimental effects.

## Materials and methods

### Ethics statement

All animal studies were conducted in accordance with the Guide for Care and Use of Laboratory Animals of the National Institutes of Health and approved by the Saint Louis University Animal Care and Use Committee (IACUC protocol # 2667).

### Viruses and cells

ZIKV (strain PRVABC59) was obtained from BEI (catalog No.: NR-50240) and passaged once in Vero cells (African green monkey kidney epithelial cells) purchased from American Type Culture Collection (ATCC CCL-81). All viruses were titered using a standard focus forming assay (FFA) on Vero cells as previously described [66].

### Mice and infections

Wild type C57BL/6J and interferon αβ receptor 1 knockout (Ifnar1^-/-^) mice were purchased commercially from Jackson Laboratories and housed in a pathogen-free mouse facility at the Saint Louis University School of Medicine. For CD4^+^T cell depletion studies, 10- to 12-week-old Ifnar1^-/-^ mice were infected subcutaneously (SC) via footpad injection with 10^5^ FFU of ZIKV. For epitope identification, wild type C57BL/6J mice were infected intravenously (IV) with 10^6^ FFU of virus. For CD4^+^T cell adoptive transfer studies, 10-12-week-old Ifnar1^-/-^ mice were infected IV with 10^5^ FFU of ZIKV one day after adoptive transfer of cells. During the course of infection mice were assessed for weight loss, signs of neurological disease, and mortality daily. Signs of disease range and in the most severe cases accelerate in the following manner from no apparent disease, limp tail, hind limb weakness, hind limb paralysis, complete paralysis and death. Occasionally mice will display multiple signs of disease at once, such as limp tail accompanied by hind limb weakness. In such instances, mice are scored as the more severe sign of disease (e.g. hind limb weakness).

### CD4^+^T cell depletion

Ifnar1^-/-^ mice were treated with 100 μg of CD4-depleting antibody clone GK1.5, two times; once on day -3 and again on day 0. To confirm CD4^+^T cell depletion blood was collected by cheek bleed on day 4 post-infection and analyzed by flow cytometry using the following antibodies: α-CD4-APC-Cy7 (clone RM4-5), α-CD8- PerCP-Cy 5.5 (clone 53–6.7), α-CD3-AF700 (clone 500A2), and α-CD19- BV605 (clone 1D3). We confirmed that CD4^+^T cell depletion was maintained through the acute phase of infection.

### Measurement of viral burden

On days 4 and 8 post-infection, intracardiac perfusion (20 ml of PBS) was performed and organs were recovered. Blood was collected in EDTA coated tubes. Organs were harvested in 1.5 ml Eppendorf tubes and snap frozen, weighed, and homogenized in DMEM using a BeadMill 24 from Fisher scientific. Viral RNA was extracted from the organ lysates using TriReagent RT. Viral RNA was extracted from whole blood using RNAzol BD. Viral RNA was quantified by qRT-PCR using Prime-Time primer-probe sets from IDT with the following sequences: Forward- CCGCTGCCCAACACAAG, Reverse- CCACTAACGTTCTTTTGCAGACAT, Probe- AGCCTACCTTGACAAGCAGTCAGACACTCAA.

### Hematology

On days 4 and 8 post-infection, mice were terminally bled into EDTA tubes and hematological parameters were assessed using an IDEXX ProCyte Dx hematology machine.

### Histological procedures

On day 8 post-infection, intracardiac perfusion was performed with 20 ml of PBS, followed by 5 ml of 4% paraformaldehyde. Brains were harvested into a 4% paraformaldehyde solution prior to embedding in paraffin blocks for sectioning. Sections were mounted, processed and stained with haematoxylin and eosin (H&E) to observe lesions. A blinded licensed pathologist analyzed the slides for signs of inflammation.

### Peptide library

A complete ZIKV peptide library was constructed based on amino acid sequences from ZIKV-PRVABC59 (BEI catalog No.: NR-50240). The library consists of 683 15-mer peptides, overlapping by 10 amino acids (S1 Table). Together, it spans the entire polyprotein. Lyophilized peptides were reconstituted to 10 mg/ml in 90% DMSO and stored at -80°C. until use. Solubility of the individual peptides is assumed to vary across the library, however we did not identify any peptides that appeared to be completely insoluble. For epitope identification the peptides in the library was diluted such that the final concentration of each peptide was ~ 2μM.

### Peptide stimulation

Splenocytes were harvested from mice 10 post-infection for acute experiments or >30 days post infection for assessment of memory responses. Spleens were ground over a 100 μm cell strainer and suspended in RPMI with 10% FBS and HEPES. 10^6^ cells were plated per well in a round-bottom 96-well plate and stimulated for 6 hours at 37°C, 5% CO_2_ in the presence of 10 μg/ml brefeldin A and either α-CD3 (clone 2C11) as a positive control or 10 μg of peptide.

### Flow cytometry

Following peptide stimulation, cells were washed with PBS and stained for the following surface markers: α-CD4-APC-Cy7 (clone RM4-5), α-CD8- PerCP-Cy 5.5 (clone 53–6.7), α-CD3-AF700 (clone 500A2), and α-CD19- BV605 (clone 1D3). Cells were then fixed and permeablized and stained for the following intracellular markers: α-IFNγ- APC (clone B27) and α-TNFα- PE (clone Mab11). The cells were analyzed by flow cytometry using an Attune NxT or BD LSRII.

### Isolation of antigen-specific CNS-infiltrating lymphocytes

8 days post-infection, intracardiac perfusion was performed and brains were minced and harvested into a digestion buffer containing collagenase I (0.05%), DNase (10 μg/ml), and HEPES (10 mM) in HBSS. After 1 hour of digestion, cells were strained over a 100 μm cell strainer, washed, resuspended in 30% Percoll, and centrifuged to separate the myelin from the cells. The cell pellet was washed and resuspended in RPMI with 10% FBS and HEPES for functional evaluation (i.e. peptide stimulation).

### TCR sequencing

Wild type C57BL/6J mice were infected IV, IP, and SC with ~10^6^ FFU ZIKV. 10 DPI, splenocytes were harvested and stimulated with each immunodominant CD4^+^ epitope separately in the presence of brefeldin A as described in “Peptide Stimulation.” CD19-, CD8-, CD4+, IFN-γ+ cells were sorted for each peptide. All sorted samples were processed and genomic DNA purified using the QIAGEN Blood and Tissue Kit. Genomic DNA was amplified using multiplexed primers targeting all V and J gene segments. TCRβ CDR3 regions were amplified and sequenced using ImmunoSEQ. Synthetic templates mimicking natural V(D)J rearrangements were used to measure and correct potential amplification bias. CDR3 segments were annotated according to the International ImMunoGeneTics collaboration, identifying V, D, and J genes contributing to each rearrangement.

### TCR library development

Alignment of shared and non-shared TCRβ sequences was completed using ImmunoSEQ software provided by Adaptive Biotechnologies. Alignments of all TCR repertoires were used to identify public TCR sequences. TCR sequence relationships were visualized using Circo plots developed as described in [45].

### Adoptive transfer of CD4^+^T cells

Ten to 12-week-old WT C57BL/6J mice were injected IV with 10^5^ FFU of ZIKV or PBS as a negative control. 30 DPI, splenocytes were harvested and prepared for use in a Milteyni enrichment kit. CD4^+^T cells were purified to 97% purity using a Miltenyi negative selection kit (139-104-454) according to manufacturer’s instructions. ~3x10^6^ cells were administered to Ifnar1^-/-^ mice IV route 1 day prior to infection.

## Supporting information

S1 Fig CNS damage associated with CD4^+^ T cell depletion. Brain cross-sections of non-depleted ZIKV infected (n = 4) **(A),** CD4 depleted ZIKV infected (n = 5) **(B),** and non-infected (n = 1) **(C)** mice. Eight days post-infection, mice were perfused with PBS followed by 4% paraformaldehyde. Brains were collected, sectioned, and stained with H&E. Black arrows indicate the presence of perineuronal inflammation, red arrows indicate the presence of perivascular inflammation and black boxes indicate the presence of suspected apoptotic or karyorrhectic debris. Representative images from a single mouse from each group were taken at 400X magnification.
(TIF)

S2 Fig CD4^+^ T cell depletion in 4 week-old mice. **(A)** Survival of four-week-old Ifnar1^-/-^ mice following CD4^+^ T cell depletion and inoculation with ZIKV via footpad injection. (n = 11 control, n = 12 depleted). On day -3 and day 0, mice were administered 100 μg of depleting antibody anti-CD4 or isotype control intraperitoneally (n = 11 control, n = 12 depleted). Survival differences were statistically significant as determined using a Mantel-Cox test (p = 0.002). **(B)** Weight loss during acute ZIKV infection of four-week-old Ifnar1^-/-^ mice. As a measure of disease, mice were weighed daily for 14 days (or until death). **(C)** Neurological sequela associated with acute ZIKV infection. Mice were evaluated for signs of neurological disease daily and graphed on each day as a percentage of mice displaying that disease indicator. Signs of disease range from no apparent disease, limp tail, hind limb weakness, hind limb paralysis, complete paralysis and death. (n = 11 control, n = 12 depleted) **(D-I).** Viral burden in the peripheral and CNS tissues after CD4^+^ depletion and ZIKV infection of 4-week-old Ifnar1^-/-^ mice. CD4^+^ depleted or control mice were infected with 10^4^ FFU ZIKV via footpad injection. On day 4 (n = 7 per group) or day 7 (n = 6–7 per group) post-infection organs were harvested, snap frozen, weighed, and homogenized. Levels of viral RNA were quantified by qPCR in whole blood **(C)**, liver **(D)**, spleen **(E)**, kidney **(F)**, spinal cord **(G)**, and brain **(H)**. Data are shown as Log_10_ focus-forming unit equivalents (eq.) (as determined by standard curve) per gram or ml of tissue or blood respectively. Differences in viral titers between the depleted and non-depleted groups in all organs on both days were not statistically significant as determined by Mann-Whitney test. Data is pooled from 2 independent experiments.
(TIF)

S1 Table Full length ZIKV peptide library. A ZIKV peptide library was constructed using amino acid sequences from ZIKV strain PRVABC59 (BEI catalog No.: NR-50240). The library consists of 683 15-mer peptides, overlapping by 10 amino acids, spanning the entire polyprotein. Each peptide is given a unique number from 1 to 683 before assignment as an epitope.
(DOCX)

S2 Table Amino acid conservation of immunodominant CD4^+^ epitopes across different ZIKV lineages and strains. Amino acid residues at 15-mer loci PrM251, E646, NS1811, and NS53211 from different strains of ZIKV were compared to that of the reference library (PRVABC59). Three strains of Asian lineage were compared including R103451 (GenBank:KX694534), P6-740 (GenBank:KX377336), and FLR (GenBank:KU820897). Three strains of African lineage were also compared including MR766 (GenBank:KX377335), DAK AR (GenBank:KY348860), and IbH (GenBank:KU963574). Residues that differ from the reference sequence for the library (PRVABC59) are highlighted in grey and written in red.
(DOCX)
