# Supplementary figures and images for: CD4+T cells mediate protection against Zika associated severe disease in a mouse model of infection
Source: PLoS Pathog. 2018 Sep 13;14(9):e1007237. doi: 10.1371/journal.ppat.1007237 (PMC6136803; doi:10.1371/journal.ppat.1007237)

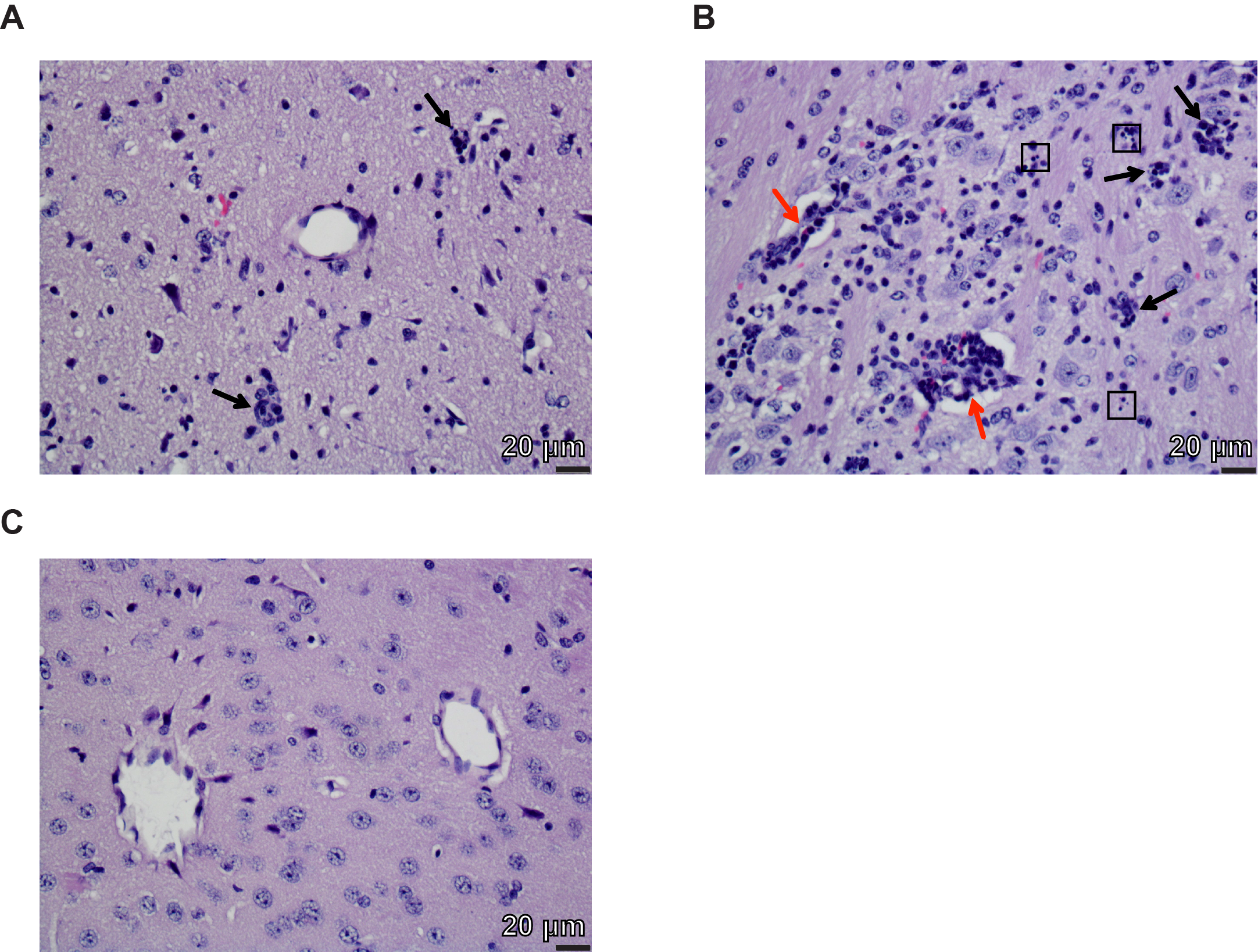

Supplement: S1 Fig — Brain cross-sections of non-depleted ZIKV infected (n = 4) (A), CD4 depleted ZIKV infected (n = 5) (B), and non-infected (n = 1) (C) mice. Eight days post-infection, mice were perfused with PBS followed by 4% paraformaldehyde. Brains were collected, sectioned, and stained with H&E. Black arrows indicate the presence of perineuronal inflammation, red arrows indicate the presence of perivascular inflammation and black boxes indicate the presence of suspected apoptotic or karyorrhectic debris. Representative images from a single mouse from each group were taken at 400X magnification. (TIF) [file ppat.1007237.s001.tif]

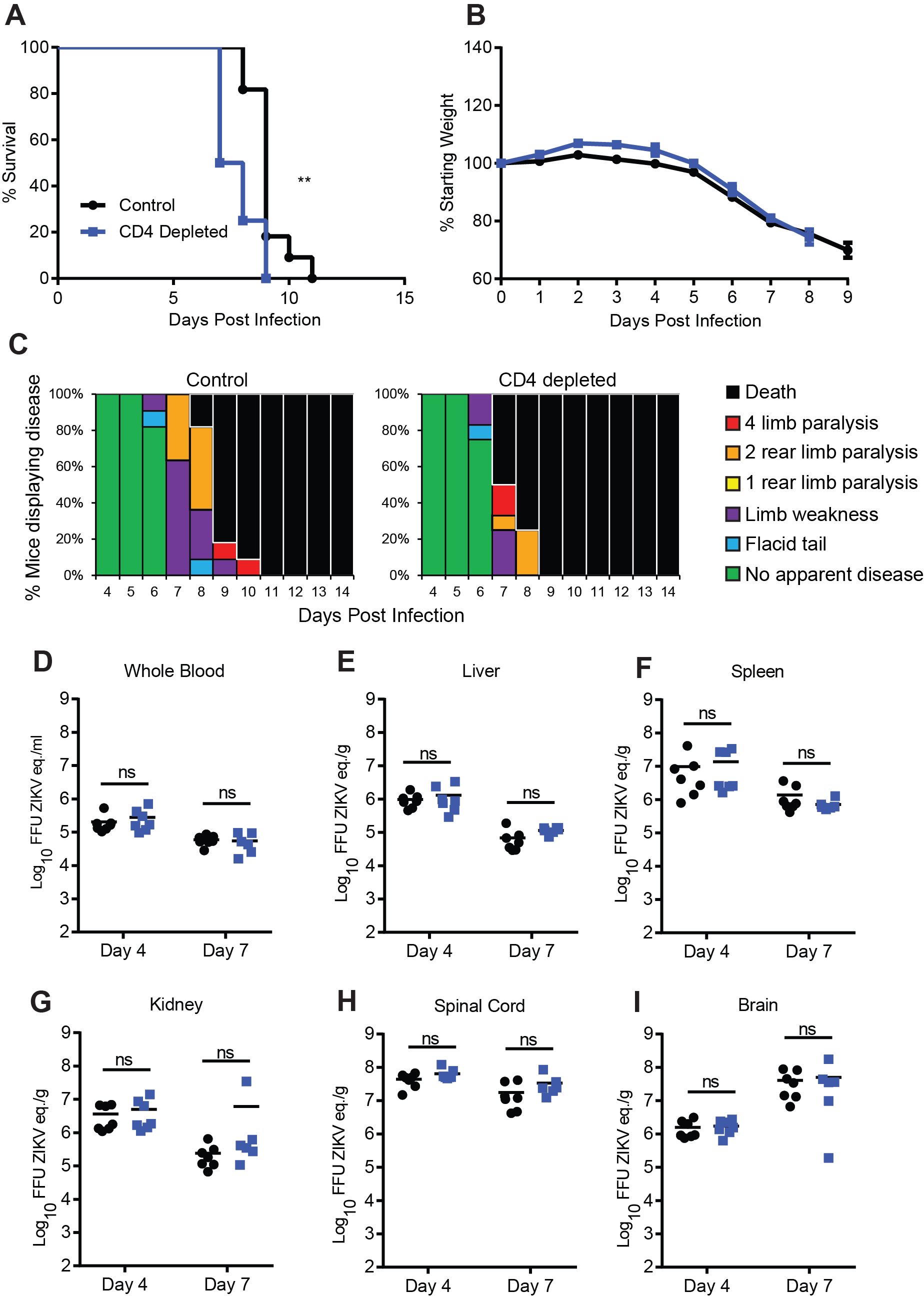

Supplement: S2 Fig — (A) Survival of four-week-old Ifnar1-/- mice following CD4+ T cell depletion and inoculation with ZIKV via footpad injection. (n = 11 control, n = 12 depleted). On day -3 and day 0, mice were administered 100 μg of depleting antibody anti-CD4 or isotype control intraperitoneally (n = 11 control, n = 12 depleted). Survival differences were statistically significant as determined using a Mantel-Cox test (p = 0.002). (B) Weight loss during acute ZIKV infection of four-week-old Ifnar1-/- mice. As a measure of disease, mice were weighed daily for 14 days (or until death). (C) Neurological sequela associated with acute ZIKV infection. Mice were evaluated for signs of neurological disease daily and graphed on each day as a percentage of mice displaying that disease indicator. Signs of disease range from no apparent disease, limp tail, hind limb weakness, hind limb paralysis, complete paralysis and death. (n = 11 control, n = 12 depleted) (D-I). Viral burden in the peripheral and CNS tissues after CD4+ depletion and ZIKV infection of 4-week-old Ifnar1-/- mice. CD4+ depleted or control mice were infected with 104 FFU ZIKV via footpad injection. On day 4 (n = 7 per group) or day 7 (n = 6–7 per group) post-infection organs were harvested, snap frozen, weighed, and homogenized. Levels of viral RNA were quantified by qPCR in whole blood (C), liver (D), spleen (E), kidney (F), spinal cord (G), and brain (H). Data are shown as Log10 focus-forming unit equivalents (eq.) (as determined by standard curve) per gram or ml of tissue or blood respectively. Differences in viral titers between the depleted and non-depleted groups in all organs on both days were not statistically significant as determined by Mann-Whitney test. Data is pooled from 2 independent experiments. (TIF) [file ppat.1007237.s002.tif]
