# Supplementary material for: CD4+T cells mediate protection against Zika associated severe disease in a mouse model of infection
Source: PLoS Pathog. 2018 Sep 13;14(9):e1007237. doi: 10.1371/journal.ppat.1007237 (PMC6136803; doi:10.1371/journal.ppat.1007237)
Supplement: S1 Table — A ZIKV peptide library was constructed using amino acid sequences from ZIKV strain PRVABC59 (BEI catalog No.: NR-50240). The library consists of 683 15-mer peptides, overlapping by 10 amino acids, spanning the entire polyprotein. Each peptide is given a unique number from 1 to 683 before assignment as an epitope. (DOCX) [file ppat.1007237.s003.docx]

| Peptide Name | Sequence |  | Peptide Name | Sequence |  | Peptide Name | Sequence |
| --- | --- | --- | --- | --- | --- | --- | --- |
| ZIKV1 | [H]MKNPKKKSGGFRIVN[OH] |  | ZIKV62 | [H]SGGTWVDVVLEHGGC[OH] |  | ZIKV123 | [H]TLHGTVTVEVQYAGT[OH] |
| ZIKV2 | [H]KKSGGFRIVNMLKRG[OH] |  | ZIKV63 | [H]VDVVLEHGGCVTVMA[OH] |  | ZIKV124 | [H]VTVEVQYAGTDGPCK[OH] |
| ZIKV3 | [H]FRIVNMLKRGVARVS[OH] |  | ZIKV64 | [H]EHGGCVTVMAQDKPT[OH] |  | ZIKV125 | [H]QYAGTDGPCKVPAQM[OH] |
| ZIKV4 | [H]MLKRGVARVSPFGGL[OH] |  | ZIKV65 | [H]VTVMAQDKPTVDIEL[OH] |  | ZIKV126 | [H]DGPCKVPAQMAVDMQ[OH] |
| ZIKV5 | [H]VARVSPFGGLKRLPA[OH] |  | ZIKV66 | [H]QDKPTVDIELVTTTV[OH] |  | ZIKV127 | [H]VPAQMAVDMQTLTPV[OH] |
| ZIKV6 | [H]PFGGLKRLPAGLLLG[OH] |  | ZIKV67 | [H]VDIELVTTTVSNMAE[OH] |  | ZIKV128 | [H]AVDMQTLTPVGRLIT[OH] |
| ZIKV7 | [H]KRLPAGLLLGHGPIR[OH] |  | ZIKV68 | [H]VTTTVSNMAEVRSYC[OH] |  | ZIKV129 | [H]TLTPVGRLITANPVI[OH] |
| ZIKV8 | [H]GLLLGHGPIRMVLAI[OH] |  | ZIKV69 | [H]SNMAEVRSYCYEASI[OH] |  | ZIKV130 | [H]GRLITANPVITESTE[OH] |
| ZIKV9 | [H]HGPIRMVLAILAFLR[OH] |  | ZIKV70 | [H]VRSYCYEASISDMAS[OH] |  | ZIKV131 | [H]ANPVITESTENSKMM[OH] |
| ZIKV10 | [H]MVLAILAFLRFTAIK[OH] |  | ZIKV71 | [H]YEASISDMASDSRCP[OH] |  | ZIKV132 | [H]TESTENSKMMLELDP[OH] |
| ZIKV11 | [H]LAFLRFTAIKPSLGL[OH] |  | ZIKV72 | [H]SDMASDSRCPTQGEA[OH] |  | ZIKV133 | [H]NSKMMLELDPPFGDS[OH] |
| ZIKV12 | [H]FTAIKPSLGLINRWG[OH] |  | ZIKV73 | [H]DSRCPTQGEAYLDKQ[OH] |  | ZIKV134 | [H]LELDPPFGDSYIVIG[OH] |
| ZIKV13 | [H]PSLGLINRWGSVGKK[OH] |  | ZIKV74 | [H]TQGEAYLDKQSDTQY[OH] |  | ZIKV135 | [H]PFGDSYIVIGVGEKK[OH] |
| ZIKV14 | [H]INRWGSVGKKEAMET[OH] |  | ZIKV75 | [H]YLDKQSDTQYVCKRT[OH] |  | ZIKV136 | [H]YIVIGVGEKKITHHW[OH] |
| ZIKV15 | [H]SVGKKEAMETIKKFK[OH] |  | ZIKV76 | [H]SDTQYVCKRTLVDRG[OH] |  | ZIKV137 | [H]VGEKKITHHWHRSGS[OH] |
| ZIKV16 | [H]EAMETIKKFKKDLAA[OH] |  | ZIKV77 | [H]VCKRTLVDRGWGNGC[OH] |  | ZIKV138 | [H]ITHHWHRSGSTIGKA[OH] |
| ZIKV17 | [H]IKKFKKDLAAMLRII[OH] |  | ZIKV78 | [H]LVDRGWGNGCGLFGK[OH] |  | ZIKV139 | [H]HRSGSTIGKAFEATV[OH] |
| ZIKV18 | [H]KDLAAMLRIINARKE[OH] |  | ZIKV79 | [H]WGNGCGLFGKGSLVT[OH] |  | ZIKV140 | [H]TIGKAFEATVRGAKR[OH] |
| ZIKV19 | [H]MLRIINARKEKKRRG[OH] |  | ZIKV80 | [H]GLFGKGSLVTCAKFA[OH] |  | ZIKV141 | [H]FEATVRGAKRMAVLG[OH] |
| ZIKV20 | [H]NARKEKKRRGADTSV[OH] |  | ZIKV81 | [H]GSLVTCAKFACSKKM[OH] |  | ZIKV142 | [H]RGAKRMAVLGDTAWD[OH] |
| ZIKV21 | [H]KKRRGADTSVGIVGL[OH] |  | ZIKV82 | [H]CAKFACSKKMTGKSI[OH] |  | ZIKV143 | [H]MAVLGDTAWDFGSVG[OH] |
| ZIKV22 | [H]ADTSVGIVGLLLTTA[OH] |  | ZIKV83 | [H]CSKKMTGKSIQPENL[OH] |  | ZIKV144 | [H]DTAWDFGSVGGALNS[OH] |
| ZIKV23 | [H]GIVGLLLTTAMAAEV[OH] |  | ZIKV84 | [H]TGKSIQPENLEYRIM[OH] |  | ZIKV145 | [H]FGSVGGALNSLGKGI[OH] |
| ZIKV24 | [H]LLTTAMAAEVTRRGS[OH] |  | ZIKV85 | [H]QPENLEYRIMLSVHG[OH] |  | ZIKV146 | [H]GALNSLGKGIHQIFG[OH] |
| ZIKV25 | [H]MAAEVTRRGSAYYMY[OH] |  | ZIKV86 | [H]EYRIMLSVHGSQHSG[OH] |  | ZIKV147 | [H]LGKGIHQIFGAAFKS[OH] |
| ZIKV26 | [H]TRRGSAYYMYLDRND[OH] |  | ZIKV87 | [H]LSVHGSQHSGMIVND[OH] |  | ZIKV148 | [H]HQIFGAAFKSLFGGM[OH] |
| ZIKV27 | [H]AYYMYLDRNDAGEAI[OH] |  | ZIKV88 | [H]SQHSGMIVNDTGHET[OH] |  | ZIKV149 | [H]AAFKSLFGGMSWFSQ[OH] |
| ZIKV28 | [H]LDRNDAGEAISFPTT[OH] |  | ZIKV89 | [H]MIVNDTGHETDENRA[OH] |  | ZIKV150 | [H]LFGGMSWFSQILIGT[OH] |
| ZIKV29 | [H]AGEAISFPTTLGMNK[OH] |  | ZIKV90 | [H]TGHETDENRAKVEIT[OH] |  | ZIKV151 | [H]SWFSQILIGTLLMWL[OH] |
| ZIKV30 | [H]SFPTTLGMNKCYIQI[OH] |  | ZIKV91 | [H]DENRAKVEITPNSPR[OH] |  | ZIKV152 | [H]ILIGTLLMWLGLNTK[OH] |
| ZIKV31 | [H]LGMNKCYIQIMDLGH[OH] |  | ZIKV92 | [H]KVEITPNSPRAEATL[OH] |  | ZIKV153 | [H]LLMWLGLNTKNGSIS[OH] |
| ZIKV32 | [H]CYIQIMDLGHMCDAT[OH] |  | ZIKV93 | [H]PNSPRAEATLGGFGS[OH] |  | ZIKV154 | [H]GLNTKNGSISLMCLA[OH] |
| ZIKV33 | [H]MDLGHMCDATMSYEC[OH] |  | ZIKV94 | [H]AEATLGGFGSLGLDC[OH] |  | ZIKV155 | [H]NGSISLMCLALGGVL[OH] |
| ZIKV34 | [H]MCDATMSYECPMLDE[OH] |  | ZIKV95 | [H]GGFGSLGLDCEPRTG[OH] |  | ZIKV156 | [H]LMCLALGGVLIFLST[OH] |
| ZIKV35 | [H]MSYECPMLDEGVEPD[OH] |  | ZIKV96 | [H]LGLDCEPRTGLDFSD[OH] |  | ZIKV157 | [H]LGGVLIFLSTAVSAD[OH] |
| ZIKV36 | [H]PMLDEGVEPDDVDCW[OH] |  | ZIKV97 | [H]EPRTGLDFSDLYYLT[OH] |  | ZIKV158 | [H]IFLSTAVSADVGCSV[OH] |
| ZIKV37 | [H]GVEPDDVDCWCNTTS[OH] |  | ZIKV98 | [H]LDFSDLYYLTMNNKH[OH] |  | ZIKV159 | [H]AVSADVGCSVDFSKK[OH] |
| ZIKV38 | [H]DVDCWCNTTSTWVVY[OH] |  | ZIKV99 | [H]LYYLTMNNKHWLVHK[OH] |  | ZIKV160 | [H]VGCSVDFSKKETRCG[OH] |
| ZIKV39 | [H]CNTTSTWVVYGTCHH[OH] |  | ZIKV100 | [H]MNNKHWLVHKEWFHD[OH] |  | ZIKV161 | [H]DFSKKETRCGTGVFV[OH] |
| ZIKV40 | [H]TWVVYGTCHHKKGEA[OH] |  | ZIKV101 | [H]WLVHKEWFHDIPLPW[OH] |  | ZIKV162 | [H]ETRCGTGVFVYNDVE[OH] |
| ZIKV41 | [H]GTCHHKKGEARRSRR[OH] |  | ZIKV102 | [H]EWFHDIPLPWHAGAD[OH] |  | ZIKV163 | [H]TGVFVYNDVEAWRDR[OH] |
| ZIKV42 | [H]KKGEARRSRRAVTLP[OH] |  | ZIKV103 | [H]IPLPWHAGADTGTPH[OH] |  | ZIKV164 | [H]YNDVEAWRDRYKYHP[OH] |
| ZIKV43 | [H]RRSRRAVTLPSHSTR[OH] |  | ZIKV104 | [H]HAGADTGTPHWNNKE[OH] |  | ZIKV165 | [H]AWRDRYKYHPDSPRR[OH] |
| ZIKV44 | [H]AVTLPSHSTRKLQTR[OH] |  | ZIKV105 | [H]TGTPHWNNKEALVEF[OH] |  | ZIKV166 | [H]YKYHPDSPRRLAAAV[OH] |
| ZIKV45 | [H]SHSTRKLQTRSQTWL[OH] |  | ZIKV106 | [H]WNNKEALVEFKDAHA[OH] |  | ZIKV167 | [H]DSPRRLAAAVKQAWE[OH] |
| ZIKV46 | [H]KLQTRSQTWLESREY[OH] |  | ZIKV107 | [H]ALVEFKDAHAKRQTV[OH] |  | ZIKV168 | [H]LAAAVKQAWEDGICG[OH] |
| ZIKV47 | [H]SQTWLESREYTKHLI[OH] |  | ZIKV108 | [H]KDAHAKRQTVVVLGS[OH] |  | ZIKV169 | [H]KQAWEDGICGISSVS[OH] |
| ZIKV48 | [H]ESREYTKHLIRVENW[OH] |  | ZIKV109 | [H]KRQTVVVLGSQEGAV[OH] |  | ZIKV170 | [H]DGICGISSVSRMENI[OH] |
| ZIKV49 | [H]TKHLIRVENWIFRNP[OH] |  | ZIKV110 | [H]VVLGSQEGAVHTALA[OH] |  | ZIKV171 | [H]ISSVSRMENIMWRSV[OH] |
| ZIKV50 | [H]RVENWIFRNPGFALA[OH] |  | ZIKV111 | [H]QEGAVHTALAGALEA[OH] |  | ZIKV172 | [H]RMENIMWRSVEGELN[OH] |
| ZIKV51 | [H]IFRNPGFALAAAAIA[OH] |  | ZIKV112 | [H]HTALAGALEAEMDGA[OH] |  | ZIKV173 | [H]MWRSVEGELNAILEE[OH] |
| ZIKV52 | [H]GFALAAAAIAWLLGS[OH] |  | ZIKV113 | [H]GALEAEMDGAKGRLS[OH] |  | ZIKV174 | [H]EGELNAILEENGVQL[OH] |
| ZIKV53 | [H]AAAIAWLLGSSTSQK[OH] |  | ZIKV114 | [H]EMDGAKGRLSSGHLK[OH] |  | ZIKV175 | [H]AILEENGVQLTVVVG[OH] |
| ZIKV54 | [H]WLLGSSTSQKVIYLV[OH] |  | ZIKV115 | [H]KGRLSSGHLKCRLKM[OH] |  | ZIKV176 | [H]NGVQLTVVVGSVKNP[OH] |
| ZIKV55 | [H]STSQKVIYLVMILLI[OH] |  | ZIKV116 | [H]SGHLKCRLKMDKLRL[OH] |  | ZIKV177 | [H]TVVVGSVKNPMWRGP[OH] |
| ZIKV56 | [H]VIYLVMILLIAPAYS[OH] |  | ZIKV117 | [H]CRLKMDKLRLKGVSY[OH] |  | ZIKV178 | [H]SVKNPMWRGPQRLPV[OH] |
| ZIKV57 | [H]MILLIAPAYSIRCIG[OH] |  | ZIKV118 | [H]DKLRLKGVSYSLCTA[OH] |  | ZIKV179 | [H]MWRGPQRLPVPVNEL[OH] |
| ZIKV58 | [H]APAYSIRCIGVSNRD[OH] |  | ZIKV119 | [H]KGVSYSLCTAAFTFT[OH] |  | ZIKV180 | [H]QRLPVPVNELPHGWK[OH] |
| ZIKV59 | [H]IRCIGVSNRDFVEGM[OH] |  | ZIKV120 | [H]SLCTAAFTFTKIPAE[OH] |  | ZIKV181 | [H]PVNELPHGWKAWGKS[OH] |
| ZIKV60 | [H]VSNRDFVEGMSGGTW[OH] |  | ZIKV121 | [H]AFTFTKIPAETLHGT[OH] |  | ZIKV182 | [H]PHGWKAWGKSYFVRA[OH] |
| ZIKV61 | [H]FVEGMSGGTWVDVVL[OH] |  | ZIKV122 | [H]KIPAETLHGTVTVEV[OH] |  | ZIKV183 | [H]AWGKSYFVRAAKTNN[OH] |
| Peptide Name | Sequence |  | Peptide Name | Sequence |  | Peptide Name | Sequence |
| ZIKV184 | [H]YFVRAAKTNNSFVVD[OH] |  | ZIKV245 | [H]AHLALIAAFKVRPAL[OH] |  | ZIKV306 | [H]RVMTRRLLGSTQVGV[OH] |
| ZIKV185 | [H]AKTNNSFVVDGDTLK[OH] |  | ZIKV246 | [H]IAAFKVRPALLVSFI[OH] |  | ZIKV307 | [H]RLLGSTQVGVGVMQE[OH] |
| ZIKV186 | [H]SFVVDGDTLKECPLK[OH] |  | ZIKV247 | [H]VRPALLVSFIFRANW[OH] |  | ZIKV308 | [H]TQVGVGVMQEGVFHT[OH] |
| ZIKV187 | [H]GDTLKECPLKHRAWN[OH] |  | ZIKV248 | [H]LVSFIFRANWTPRES[OH] |  | ZIKV309 | [H]GVMQEGVFHTMWHVT[OH] |
| ZIKV188 | [H]ECPLKHRAWNSFLVE[OH] |  | ZIKV249 | [H]FRANWTPRESMLLAL[OH] |  | ZIKV310 | [H]GVFHTMWHVTKGSAL[OH] |
| ZIKV189 | [H]HRAWNSFLVEDHGFG[OH] |  | ZIKV250 | [H]TPRESMLLALASCLL[OH] |  | ZIKV311 | [H]MWHVTKGSALRSGEG[OH] |
| ZIKV190 | [H]SFLVEDHGFGVFHTS[OH] |  | ZIKV251 | [H]MLLALASCLLQTAIS[OH] |  | ZIKV312 | [H]KGSALRSGEGRLDPY[OH] |
| ZIKV191 | [H]DHGFGVFHTSVWLKV[OH] |  | ZIKV252 | [H]ASCLLQTAISALEGD[OH] |  | ZIKV313 | [H]RSGEGRLDPYWGDVK[OH] |
| ZIKV192 | [H]VFHTSVWLKVREDYS[OH] |  | ZIKV253 | [H]QTAISALEGDLMVLI[OH] |  | ZIKV314 | [H]RLDPYWGDVKQDLVS[OH] |
| ZIKV193 | [H]VWLKVREDYSLECDP[OH] |  | ZIKV254 | [H]ALEGDLMVLINGFAL[OH] |  | ZIKV315 | [H]WGDVKQDLVSYCGPW[OH] |
| ZIKV194 | [H]REDYSLECDPAVIGT[OH] |  | ZIKV255 | [H]LMVLINGFALAWLAI[OH] |  | ZIKV316 | [H]QDLVSYCGPWKLDAA[OH] |
| ZIKV195 | [H]LECDPAVIGTAVKGK[OH] |  | ZIKV256 | [H]NGFALAWLAIRAMVV[OH] |  | ZIKV317 | [H]YCGPWKLDAAWDGHS[OH] |
| ZIKV196 | [H]AVIGTAVKGKEAVHS[OH] |  | ZIKV257 | [H]AWLAIRAMVVPRTDN[OH] |  | ZIKV318 | [H]KLDAAWDGHSEVQLL[OH] |
| ZIKV197 | [H]AVKGKEAVHSDLGYW[OH] |  | ZIKV258 | [H]RAMVVPRTDNITLAI[OH] |  | ZIKV319 | [H]WDGHSEVQLLAVPPG[OH] |
| ZIKV198 | [H]EAVHSDLGYWIESEK[OH] |  | ZIKV259 | [H]PRTDNITLAILAALT[OH] |  | ZIKV320 | [H]EVQLLAVPPGERARN[OH] |
| ZIKV199 | [H]DLGYWIESEKNDTWR[OH] |  | ZIKV260 | [H]ITLAILAALTPLARG[OH] |  | ZIKV321 | [H]AVPPGERARNIQTLP[OH] |
| ZIKV200 | [H]IESEKNDTWRLKRAH[OH] |  | ZIKV261 | [H]LAALTPLARGTLLVA[OH] |  | ZIKV322 | [H]ERARNIQTLPGIFKT[OH] |
| ZIKV201 | [H]NDTWRLKRAHLIEMK[OH] |  | ZIKV262 | [H]PLARGTLLVAWRAGL[OH] |  | ZIKV323 | [H]IQTLPGIFKTKDGDI[OH] |
| ZIKV202 | [H]LKRAHLIEMKTCEWP[OH] |  | ZIKV263 | [H]TLLVAWRAGLATCGG[OH] |  | ZIKV324 | [H]GIFKTKDGDIGAVAL[OH] |
| ZIKV203 | [H]LIEMKTCEWPKSHTL[OH] |  | ZIKV264 | [H]WRAGLATCGGFMLLS[OH] |  | ZIKV325 | [H]KDGDIGAVALDYPAG[OH] |
| ZIKV204 | [H]TCEWPKSHTLWTDGI[OH] |  | ZIKV265 | [H]ATCGGFMLLSLKGKG[OH] |  | ZIKV326 | [H]GAVALDYPAGTSGSP[OH] |
| ZIKV205 | [H]KSHTLWTDGIEESDL[OH] |  | ZIKV266 | [H]FMLLSLKGKGSVKKN[OH] |  | ZIKV327 | [H]DYPAGTSGSPILDKC[OH] |
| ZIKV206 | [H]WTDGIEESDLIIPKS[OH] |  | ZIKV267 | [H]LKGKGSVKKNLPFVM[OH] |  | ZIKV328 | [H]TSGSPILDKCGRVIG[OH] |
| ZIKV207 | [H]EESDLIIPKSLAGPL[OH] |  | ZIKV268 | [H]SVKKNLPFVMALGLT[OH] |  | ZIKV329 | [H]ILDKCGRVIGLYGNG[OH] |
| ZIKV208 | [H]IIPKSLAGPLSHHNT[OH] |  | ZIKV269 | [H]LPFVMALGLTAVRLV[OH] |  | ZIKV330 | [H]GRVIGLYGNGVVIKN[OH] |
| ZIKV209 | [H]LAGPLSHHNTREGYR[OH] |  | ZIKV270 | [H]ALGLTAVRLVDPINV[OH] |  | ZIKV331 | [H]LYGNGVVIKNGSYVS[OH] |
| ZIKV210 | [H]SHHNTREGYRTQMKG[OH] |  | ZIKV271 | [H]AVRLVDPINVVGLLL[OH] |  | ZIKV332 | [H]VVIKNGSYVSAITQG[OH] |
| ZIKV211 | [H]REGYRTQMKGPWHSE[OH] |  | ZIKV272 | [H]DPINVVGLLLLTRSG[OH] |  | ZIKV333 | [H]GSYVSAITQGRREEE[OH] |
| ZIKV212 | [H]TQMKGPWHSEELEIR[OH] |  | ZIKV273 | [H]VGLLLLTRSGKRSWP[OH] |  | ZIKV334 | [H]AITQGRREEETPVEC[OH] |
| ZIKV213 | [H]PWHSEELEIRFEECP[OH] |  | ZIKV274 | [H]LTRSGKRSWPPSEVL[OH] |  | ZIKV335 | [H]RREEETPVECFEPSM[OH] |
| ZIKV214 | [H]ELEIRFEECPGTKVH[OH] |  | ZIKV275 | [H]KRSWPPSEVLTAVGL[OH] |  | ZIKV336 | [H]TPVECFEPSMLKKKQ[OH] |
| ZIKV215 | [H]FEECPGTKVHVEETC[OH] |  | ZIKV276 | [H]PSEVLTAVGLICALA[OH] |  | ZIKV337 | [H]FEPSMLKKKQLTVLD[OH] |
| ZIKV216 | [H]GTKVHVEETCGTRGP[OH] |  | ZIKV277 | [H]TAVGLICALAGGFAK[OH] |  | ZIKV338 | [H]LKKKQLTVLDLHPGA[OH] |
| ZIKV217 | [H]VEETCGTRGPSLRST[OH] |  | ZIKV278 | [H]ICALAGGFAKADIEM[OH] |  | ZIKV339 | [H]LTVLDLHPGAGKTRR[OH] |
| ZIKV218 | [H]GTRGPSLRSTTASGR[OH] |  | ZIKV279 | [H]GGFAKADIEMAGPMA[OH] |  | ZIKV340 | [H]LHPGAGKTRRVLPEI[OH] |
| ZIKV219 | [H]SLRSTTASGRVIEEW[OH] |  | ZIKV280 | [H]ADIEMAGPMAAVGLL[OH] |  | ZIKV341 | [H]GKTRRVLPEIVREAI[OH] |
| ZIKV220 | [H]TASGRVIEEWCCREC[OH] |  | ZIKV281 | [H]AGPMAAVGLLIVSYV[OH] |  | ZIKV342 | [H]VLPEIVREAIKTRLR[OH] |
| ZIKV221 | [H]VIEEWCCRECTMPPL[OH] |  | ZIKV282 | [H]AVGLLIVSYVVSGKS[OH] |  | ZIKV343 | [H]VREAIKTRLRTVILA[OH] |
| ZIKV222 | [H]CCRECTMPPLSFRAK[OH] |  | ZIKV283 | [H]IVSYVVSGKSVDMYI[OH] |  | ZIKV344 | [H]KTRLRTVILAPTRVV[OH] |
| ZIKV223 | [H]TMPPLSFRAKDGCWY[OH] |  | ZIKV284 | [H]VSGKSVDMYIERAGD[OH] |  | ZIKV345 | [H]TVILAPTRVVAAEME[OH] |
| ZIKV224 | [H]SFRAKDGCWYGMEIR[OH] |  | ZIKV285 | [H]VDMYIERAGDITWEK[OH] |  | ZIKV346 | [H]PTRVVAAEMEEALRG[OH] |
| ZIKV225 | [H]DGCWYGMEIRPRKEP[OH] |  | ZIKV286 | [H]ERAGDITWEKDAEVT[OH] |  | ZIKV347 | [H]AAEMEEALRGLPVRY[OH] |
| ZIKV226 | [H]GMEIRPRKEPESNLV[OH] |  | ZIKV287 | [H]ITWEKDAEVTGNSPR[OH] |  | ZIKV348 | [H]EALRGLPVRYMTTAV[OH] |
| ZIKV227 | [H]PRKEPESNLVRSMVT[OH] |  | ZIKV288 | [H]DAEVTGNSPRLDVAL[OH] |  | ZIKV349 | [H]LPVRYMTTAVNVTHS[OH] |
| ZIKV228 | [H]ESNLVRSMVTAGSTD[OH] |  | ZIKV289 | [H]GNSPRLDVALDESGD[OH] |  | ZIKV350 | [H]MTTAVNVTHSGTEIV[OH] |
| ZIKV229 | [H]RSMVTAGSTDHMDHF[OH] |  | ZIKV290 | [H]LDVALDESGDFSLVE[OH] |  | ZIKV351 | [H]NVTHSGTEIVDLMCH[OH] |
| ZIKV230 | [H]AGSTDHMDHFSLGVL[OH] |  | ZIKV291 | [H]DESGDFSLVEDDGPP[OH] |  | ZIKV352 | [H]GTEIVDLMCHATFTS[OH] |
| ZIKV231 | [H]HMDHFSLGVLVILLM[OH] |  | ZIKV292 | [H]FSLVEDDGPPMREII[OH] |  | ZIKV353 | [H]DLMCHATFTSRLLQP[OH] |
| ZIKV232 | [H]SLGVLVILLMVQEGL[OH] |  | ZIKV293 | [H]DDGPPMREIILKVVL[OH] |  | ZIKV354 | [H]ATFTSRLLQPIRVPN[OH] |
| ZIKV233 | [H]VILLMVQEGLKKRMT[OH] |  | ZIKV294 | [H]MREIILKVVLMTICG[OH] |  | ZIKV355 | [H]RLLQPIRVPNYNLYI[OH] |
| ZIKV234 | [H]VQEGLKKRMTTKIII[OH] |  | ZIKV295 | [H]LKVVLMTICGMNPIA[OH] |  | ZIKV356 | [H]IRVPNYNLYIMDEAH[OH] |
| ZIKV235 | [H]KKRMTTKIIISTSMA[OH] |  | ZIKV296 | [H]MTICGMNPIAIPFAA[OH] |  | ZIKV357 | [H]YNLYIMDEAHFTDPS[OH] |
| ZIKV236 | [H]TKIIISTSMAVLVAM[OH] |  | ZIKV297 | [H]MNPIAIPFAAGAWYV[OH] |  | ZIKV358 | [H]MDEAHFTDPSSIAAR[OH] |
| ZIKV237 | [H]STSMAVLVAMILGGF[OH] |  | ZIKV298 | [H]IPFAAGAWYVYVKTG[OH] |  | ZIKV359 | [H]FTDPSSIAARGYIST[OH] |
| ZIKV238 | [H]VLVAMILGGFSMSDL[OH] |  | ZIKV299 | [H]GAWYVYVKTGKRSGA[OH] |  | ZIKV360 | [H]SIAARGYISTRVEMG[OH] |
| ZIKV239 | [H]ILGGFSMSDLAKLAI[OH] |  | ZIKV300 | [H]YVKTGKRSGALWDVP[OH] |  | ZIKV361 | [H]GYISTRVEMGEAAAI[OH] |
| ZIKV240 | [H]SMSDLAKLAILMGAT[OH] |  | ZIKV301 | [H]KRSGALWDVPAPKEV[OH] |  | ZIKV362 | [H]RVEMGEAAAIFMTAT[OH] |
| ZIKV241 | [H]AKLAILMGATFAEMN[OH] |  | ZIKV302 | [H]LWDVPAPKEVKKGET[OH] |  | ZIKV363 | [H]EAAAIFMTATPPGTR[OH] |
| ZIKV242 | [H]LMGATFAEMNTGGDV[OH] |  | ZIKV303 | [H]APKEVKKGETTDGVY[OH] |  | ZIKV364 | [H]FMTATPPGTRDAFPD[OH] |
| ZIKV243 | [H]FAEMNTGGDVAHLAL[OH] |  | ZIKV304 | [H]KKGETTDGVYRVMTR[OH] |  | ZIKV365 | [H]PPGTRDAFPDSNSPI[OH] |
| ZIKV244 | [H]TGGDVAHLALIAAFK[OH] |  | ZIKV305 | [H]TDGVYRVMTRRLLGS[OH] |  | ZIKV366 | [H]DAFPDSNSPIMDTEV[OH] |
| Peptide Name | Sequence |  | Peptide Name | Sequence |  | Peptide Name | Sequence |
| ZIKV367 | [H]SNSPIMDTEVEVPER[OH] |  | ZIKV428 | [H]MTERFQEAIDNLAVL[OH] |  | ZIKV489 | [H]QVLLIAVAVSSAILS[OH] |
| ZIKV368 | [H]MDTEVEVPERAWSSG[OH] |  | ZIKV429 | [H]QEAIDNLAVLMRAET[OH] |  | ZIKV490 | [H]AVAVSSAILSRTAWG[OH] |
| ZIKV369 | [H]EVPERAWSSGFDWVT[OH] |  | ZIKV430 | [H]NLAVLMRAETGSRPY[OH] |  | ZIKV491 | [H]SAILSRTAWGWGEAG[OH] |
| ZIKV370 | [H]AWSSGFDWVTDHSGK[OH] |  | ZIKV431 | [H]MRAETGSRPYKAAAA[OH] |  | ZIKV492 | [H]RTAWGWGEAGALITA[OH] |
| ZIKV371 | [H]FDWVTDHSGKTVWFV[OH] |  | ZIKV432 | [H]GSRPYKAAAAQLPET[OH] |  | ZIKV493 | [H]WGEAGALITAATSTL[OH] |
| ZIKV372 | [H]DHSGKTVWFVPSVRN[OH] |  | ZIKV433 | [H]KAAAAQLPETLETIM[OH] |  | ZIKV494 | [H]ALITAATSTLWEGSP[OH] |
| ZIKV373 | [H]TVWFVPSVRNGNEIA[OH] |  | ZIKV434 | [H]QLPETLETIMLLGLL[OH] |  | ZIKV495 | [H]ATSTLWEGSPNKYWN[OH] |
| ZIKV374 | [H]PSVRNGNEIAACLTK[OH] |  | ZIKV435 | [H]LETIMLLGLLGTVSL[OH] |  | ZIKV496 | [H]WEGSPNKYWNSSTAT[OH] |
| ZIKV375 | [H]GNEIAACLTKAGKRV[OH] |  | ZIKV436 | [H]LLGLLGTVSLGIFFV[OH] |  | ZIKV497 | [H]NKYWNSSTATSLCNI[OH] |
| ZIKV376 | [H]ACLTKAGKRVIQLSR[OH] |  | ZIKV437 | [H]GTVSLGIFFVLMRNK[OH] |  | ZIKV498 | [H]SSTATSLCNIFRGSY[OH] |
| ZIKV377 | [H]AGKRVIQLSRKTFET[OH] |  | ZIKV438 | [H]GIFFVLMRNKGIGKM[OH] |  | ZIKV499 | [H]SLCNIFRGSYLAGAS[OH] |
| ZIKV378 | [H]IQLSRKTFETEFQKT[OH] |  | ZIKV439 | [H]LMRNKGIGKMGFGMV[OH] |  | ZIKV500 | [H]FRGSYLAGASLIYTV[OH] |
| ZIKV379 | [H]KTFETEFQKTKHQEW[OH] |  | ZIKV440 | [H]GIGKMGFGMVTLGAS[OH] |  | ZIKV501 | [H]LAGASLIYTVTRNAG[OH] |
| ZIKV380 | [H]EFQKTKHQEWDFVVT[OH] |  | ZIKV441 | [H]GFGMVTLGASAWLMW[OH] |  | ZIKV502 | [H]LIYTVTRNAGLVKRR[OH] |
| ZIKV381 | [H]KHQEWDFVVTTDISE[OH] |  | ZIKV442 | [H]TLGASAWLMWLSEIE[OH] |  | ZIKV503 | [H]TRNAGLVKRRGGGTG[OH] |
| ZIKV382 | [H]DFVVTTDISEMGANF[OH] |  | ZIKV443 | [H]AWLMWLSEIEPARIA[OH] |  | ZIKV504 | [H]LVKRRGGGTGETLGE[OH] |
| ZIKV383 | [H]TDISEMGANFKADRV[OH] |  | ZIKV444 | [H]LSEIEPARIACVLIV[OH] |  | ZIKV505 | [H]GGGTGETLGEKWKAR[OH] |
| ZIKV384 | [H]MGANFKADRVIDSRR[OH] |  | ZIKV445 | [H]PARIACVLIVVFLLL[OH] |  | ZIKV506 | [H]ETLGEKWKARLNQMS[OH] |
| ZIKV385 | [H]KADRVIDSRRCLKPV[OH] |  | ZIKV446 | [H]CVLIVVFLLLVVLIP[OH] |  | ZIKV507 | [H]KWKARLNQMSALEFY[OH] |
| ZIKV386 | [H]IDSRRCLKPVILDGE[OH] |  | ZIKV447 | [H]VFLLLVVLIPEPEKQ[OH] |  | ZIKV508 | [H]LNQMSALEFYSYKKS[OH] |
| ZIKV387 | [H]CLKPVILDGERVILA[OH] |  | ZIKV448 | [H]VVLIPEPEKQRSPQD[OH] |  | ZIKV509 | [H]ALEFYSYKKSGITEV[OH] |
| ZIKV388 | [H]ILDGERVILAGPMPV[OH] |  | ZIKV449 | [H]EPEKQRSPQDNQMAI[OH] |  | ZIKV510 | [H]SYKKSGITEVCREEA[OH] |
| ZIKV389 | [H]RVILAGPMPVTHASA[OH] |  | ZIKV450 | [H]RSPQDNQMAIIIMVA[OH] |  | ZIKV511 | [H]GITEVCREEARRALK[OH] |
| ZIKV390 | [H]GPMPVTHASAAQRRG[OH] |  | ZIKV451 | [H]NQMAIIIMVAVGLLG[OH] |  | ZIKV512 | [H]CREEARRALKDGVAT[OH] |
| ZIKV391 | [H]THASAAQRRGRIGRN[OH] |  | ZIKV452 | [H]IIMVAVGLLGLITAN[OH] |  | ZIKV513 | [H]RRALKDGVATGGHAV[OH] |
| ZIKV392 | [H]AQRRGRIGRNPNKPG[OH] |  | ZIKV453 | [H]VGLLGLITANELGWL[OH] |  | ZIKV514 | [H]DGVATGGHAVSRGSA[OH] |
| ZIKV393 | [H]RIGRNPNKPGDEYLY[OH] |  | ZIKV454 | [H]LITANELGWLERTKS[OH] |  | ZIKV515 | [H]GGHAVSRGSAKLRWL[OH] |
| ZIKV394 | [H]PNKPGDEYLYGGGCA[OH] |  | ZIKV455 | [H]ELGWLERTKSDLSHL[OH] |  | ZIKV516 | [H]SRGSAKLRWLVERGY[OH] |
| ZIKV395 | [H]DEYLYGGGCAETDED[OH] |  | ZIKV456 | [H]ERTKSDLSHLMGRRE[OH] |  | ZIKV517 | [H]KLRWLVERGYLQPYG[OH] |
| ZIKV396 | [H]GGGCAETDEDHAHWL[OH] |  | ZIKV457 | [H]DLSHLMGRREEGATI[OH] |  | ZIKV518 | [H]VERGYLQPYGKVIDL[OH] |
| ZIKV397 | [H]ETDEDHAHWLEARML[OH] |  | ZIKV458 | [H]MGRREEGATIGFSMD[OH] |  | ZIKV519 | [H]LQPYGKVIDLGCGRG[OH] |
| ZIKV398 | [H]HAHWLEARMLLDNIY[OH] |  | ZIKV459 | [H]EGATIGFSMDIDLRP[OH] |  | ZIKV520 | [H]KVIDLGCGRGGWSYY[OH] |
| ZIKV399 | [H]EARMLLDNIYLQDGL[OH] |  | ZIKV460 | [H]GFSMDIDLRPASAWA[OH] |  | ZIKV521 | [H]GCGRGGWSYYVATIR[OH] |
| ZIKV400 | [H]LDNIYLQDGLIASLY[OH] |  | ZIKV461 | [H]IDLRPASAWAIYAAL[OH] |  | ZIKV522 | [H]GWSYYVATIRKVQEV[OH] |
| ZIKV401 | [H]LQDGLIASLYRPEAD[OH] |  | ZIKV462 | [H]ASAWAIYAALTTFIT[OH] |  | ZIKV523 | [H]VATIRKVQEVKGYTK[OH] |
| ZIKV402 | [H]IASLYRPEADKVAAI[OH] |  | ZIKV463 | [H]IYAALTTFITPAVQH[OH] |  | ZIKV524 | [H]KVQEVKGYTKGGPGH[OH] |
| ZIKV403 | [H]RPEADKVAAIEGEFK[OH] |  | ZIKV464 | [H]TTFITPAVQHAVTTS[OH] |  | ZIKV525 | [H]KGYTKGGPGHEEPVL[OH] |
| ZIKV404 | [H]KVAAIEGEFKLRTEQ[OH] |  | ZIKV465 | [H]PAVQHAVTTSYNNYS[OH] |  | ZIKV526 | [H]GGPGHEEPVLVQSYG[OH] |
| ZIKV405 | [H]EGEFKLRTEQRKTFV[OH] |  | ZIKV466 | [H]AVTTSYNNYSLMAMA[OH] |  | ZIKV527 | [H]EEPVLVQSYGWNIVR[OH] |
| ZIKV406 | [H]LRTEQRKTFVELMKR[OH] |  | ZIKV467 | [H]YNNYSLMAMATQAGV[OH] |  | ZIKV528 | [H]VQSYGWNIVRLKSGV[OH] |
| ZIKV407 | [H]RKTFVELMKRGDLPV[OH] |  | ZIKV468 | [H]LMAMATQAGVLFGMG[OH] |  | ZIKV529 | [H]WNIVRLKSGVDVFHM[OH] |
| ZIKV408 | [H]ELMKRGDLPVWLAYQ[OH] |  | ZIKV469 | [H]TQAGVLFGMGKGMPF[OH] |  | ZIKV530 | [H]LKSGVDVFHMAAEPC[OH] |
| ZIKV409 | [H]GDLPVWLAYQVASAG[OH] |  | ZIKV470 | [H]LFGMGKGMPFYAWDF[OH] |  | ZIKV531 | [H]DVFHMAAEPCDTLLC[OH] |
| ZIKV410 | [H]WLAYQVASAGITYTD[OH] |  | ZIKV471 | [H]KGMPFYAWDFGVPLL[OH] |  | ZIKV532 | [H]AAEPCDTLLCDIGES[OH] |
| ZIKV411 | [H]VASAGITYTDRRWCF[OH] |  | ZIKV472 | [H]YAWDFGVPLLMIGCY[OH] |  | ZIKV533 | [H]DTLLCDIGESSSSPE[OH] |
| ZIKV412 | [H]ITYTDRRWCFDGTTN[OH] |  | ZIKV473 | [H]GVPLLMIGCYSQLTP[OH] |  | ZIKV534 | [H]DIGESSSSPEVEEAR[OH] |
| ZIKV413 | [H]RRWCFDGTTNNTIME[OH] |  | ZIKV474 | [H]MIGCYSQLTPLTLIV[OH] |  | ZIKV535 | [H]SSSPEVEEARTLRVL[OH] |
| ZIKV414 | [H]DGTTNNTIMEDSVPA[OH] |  | ZIKV475 | [H]SQLTPLTLIVAIILL[OH] |  | ZIKV536 | [H]VEEARTLRVLSMVGD[OH] |
| ZIKV415 | [H]NTIMEDSVPAEVWTR[OH] |  | ZIKV476 | [H]LTLIVAIILLVAHYM[OH] |  | ZIKV537 | [H]TLRVLSMVGDWLEKR[OH] |
| ZIKV416 | [H]DSVPAEVWTRHGEKR[OH] |  | ZIKV477 | [H]AIILLVAHYMYLIPG[OH] |  | ZIKV538 | [H]SMVGDWLEKRPGAFC[OH] |
| ZIKV417 | [H]EVWTRHGEKRVLKPR[OH] |  | ZIKV478 | [H]VAHYMYLIPGLQAAA[OH] |  | ZIKV539 | [H]WLEKRPGAFCIKVLC[OH] |
| ZIKV418 | [H]HGEKRVLKPRWMDAR[OH] |  | ZIKV479 | [H]YLIPGLQAAAARAAQ[OH] |  | ZIKV540 | [H]PGAFCIKVLCPYTST[OH] |
| ZIKV419 | [H]VLKPRWMDARVCSDH[OH] |  | ZIKV480 | [H]LQAAAARAAQKRTAA[OH] |  | ZIKV541 | [H]IKVLCPYTSTMMETL[OH] |
| ZIKV420 | [H]WMDARVCSDHAALKS[OH] |  | ZIKV481 | [H]ARAAQKRTAAGIMKN[OH] |  | ZIKV542 | [H]PYTSTMMETLERLQR[OH] |
| ZIKV421 | [H]VCSDHAALKSFKEFA[OH] |  | ZIKV482 | [H]KRTAAGIMKNPVVDG[OH] |  | ZIKV543 | [H]MMETLERLQRRYGGG[OH] |
| ZIKV422 | [H]AALKSFKEFAAGKRG[OH] |  | ZIKV483 | [H]GIMKNPVVDGIVVTD[OH] |  | ZIKV544 | [H]ERLQRRYGGGLVRVP[OH] |
| ZIKV423 | [H]FKEFAAGKRGAAFGV[OH] |  | ZIKV484 | [H]PVVDGIVVTDIDTMT[OH] |  | ZIKV545 | [H]RYGGGLVRVPLSRNS[OH] |
| ZIKV424 | [H]AGKRGAAFGVMEALG[OH] |  | ZIKV485 | [H]IVVTDIDTMTIDPQV[OH] |  | ZIKV546 | [H]LVRVPLSRNSTHEMY[OH] |
| ZIKV425 | [H]AAFGVMEALGTLPGH[OH] |  | ZIKV486 | [H]IDTMTIDPQVEKKMG[OH] |  | ZIKV547 | [H]LSRNSTHEMYWVSGA[OH] |
| ZIKV426 | [H]MEALGTLPGHMTERF[OH] |  | ZIKV487 | [H]IDPQVEKKMGQVLLI[OH] |  | ZIKV548 | [H]THEMYWVSGAKSNTI[OH] |
| ZIKV427 | [H]TLPGHMTERFQEAID[OH] |  | ZIKV488 | [H]EKKMGQVLLIAVAVS[OH] |  | ZIKV549 | [H]WVSGAKSNTIKSVST[OH] |
| Peptide Name | Sequence |  | Peptide Name | Sequence |  | Peptide Name | Sequence |
| ZIKV550 | [H]KSNTIKSVSTTSQLL[OH] |  | ZIKV611 | [H]MYADDTAGWDTRISR[OH] |  | ZIKV672 | [H]TDIPYLGKREDLWCG[OH] |
| ZIKV551 | [H]KSVSTTSQLLLGRMD[OH] |  | ZIKV612 | [H]TAGWDTRISRFDLEN[OH] |  | ZIKV673 | [H]LGKREDLWCGSLIGH[OH] |
| ZIKV552 | [H]TSQLLLGRMDGPRRP[OH] |  | ZIKV613 | [H]TRISRFDLENEALIT[OH] |  | ZIKV674 | [H]DLWCGSLIGHRPRTT[OH] |
| ZIKV553 | [H]LGRMDGPRRPVKYEE[OH] |  | ZIKV614 | [H]FDLENEALITNQMEK[OH] |  | ZIKV675 | [H]SLIGHRPRTTWAENI[OH] |
| ZIKV554 | [H]GPRRPVKYEEDVNLG[OH] |  | ZIKV615 | [H]EALITNQMEKGHRAL[OH] |  | ZIKV676 | [H]RPRTTWAENIKNTVN[OH] |
| ZIKV555 | [H]VKYEEDVNLGSGTRA[OH] |  | ZIKV616 | [H]NQMEKGHRALALAII[OH] |  | ZIKV677 | [H]WAENIKNTVNMVRRI[OH] |
| ZIKV556 | [H]DVNLGSGTRAVVSCA[OH] |  | ZIKV617 | [H]GHRALALAIIKYTYQ[OH] |  | ZIKV678 | [H]KNTVNMVRRIIGDEE[OH] |
| ZIKV557 | [H]SGTRAVVSCAEAPNM[OH] |  | ZIKV618 | [H]ALAIIKYTYQNKVVK[OH] |  | ZIKV679 | [H]MVRRIIGDEEKYMDY[OH] |
| ZIKV558 | [H]VVSCAEAPNMKIIGN[OH] |  | ZIKV619 | [H]KYTYQNKVVKVLRPA[OH] |  | ZIKV680 | [H]IGDEEKYMDYLSTQV[OH] |
| ZIKV559 | [H]EAPNMKIIGNRIERI[OH] |  | ZIKV620 | [H]NKVVKVLRPAEKGKT[OH] |  | ZIKV681 | [H]KYMDYLSTQVRYLGE[OH] |
| ZIKV560 | [H]KIIGNRIERIRSEHA[OH] |  | ZIKV621 | [H]VLRPAEKGKTVMDII[OH] |  | ZIKV682 | [H]LSTQVRYLGEEGSTP[OH] |
| ZIKV561 | [H]RIERIRSEHAETWFF[OH] |  | ZIKV622 | [H]EKGKTVMDIISRQDQ[OH] |  | ZIKV683 | [H]RYLGEEGSTPGVL[OH] |
| ZIKV562 | [H]RSEHAETWFFDENHP[OH] |  | ZIKV623 | [H]VMDIISRQDQRGSGQ[OH] |  |  |  |
| ZIKV563 | [H]ETWFFDENHPYRTWA[OH] |  | ZIKV624 | [H]SRQDQRGSGQVVTYA[OH] |  |  |  |
| ZIKV564 | [H]DENHPYRTWAYHGSY[OH] |  | ZIKV625 | [H]RGSGQVVTYALNTFT[OH] |  |  |  |
| ZIKV565 | [H]YRTWAYHGSYEAPTQ[OH] |  | ZIKV626 | [H]VVTYALNTFTNLVVQ[OH] |  |  |  |
| ZIKV566 | [H]YHGSYEAPTQGSASS[OH] |  | ZIKV627 | [H]LNTFTNLVVQLIRNM[OH] |  |  |  |
| ZIKV567 | [H]EAPTQGSASSLINGV[OH] |  | ZIKV628 | [H]NLVVQLIRNMEAEEV[OH] |  |  |  |
| ZIKV568 | [H]GSASSLINGVVRLLS[OH] |  | ZIKV629 | [H]LIRNMEAEEVLEMQD[OH] |  |  |  |
| ZIKV569 | [H]LINGVVRLLSKPWDV[OH] |  | ZIKV630 | [H]EAEEVLEMQDLWLLR[OH] |  |  |  |
| ZIKV570 | [H]VRLLSKPWDVVTGVT[OH] |  | ZIKV631 | [H]LEMQDLWLLRRSEKV[OH] |  |  |  |
| ZIKV571 | [H]KPWDVVTGVTGIAMT[OH] |  | ZIKV632 | [H]LWLLRRSEKVTNWLQ[OH] |  |  |  |
| ZIKV572 | [H]VTGVTGIAMTDTTPY[OH] |  | ZIKV633 | [H]RSEKVTNWLQSNGWD[OH] |  |  |  |
| ZIKV573 | [H]GIAMTDTTPYGQQRV[OH] |  | ZIKV634 | [H]TNWLQSNGWDRLKRM[OH] |  |  |  |
| ZIKV574 | [H]DTTPYGQQRVFKEKV[OH] |  | ZIKV635 | [H]SNGWDRLKRMAVSGD[OH] |  |  |  |
| ZIKV575 | [H]GQQRVFKEKVDTRVP[OH] |  | ZIKV636 | [H]RLKRMAVSGDDCVVK[OH] |  |  |  |
| ZIKV576 | [H]FKEKVDTRVPDPQEG[OH] |  | ZIKV637 | [H]AVSGDDCVVKPIDDR[OH] |  |  |  |
| ZIKV577 | [H]DTRVPDPQEGTRQVM[OH] |  | ZIKV638 | [H]DCVVKPIDDRFAHAL[OH] |  |  |  |
| ZIKV578 | [H]DPQEGTRQVMSMVSS[OH] |  | ZIKV639 | [H]PIDDRFAHALRFLND[OH] |  |  |  |
| ZIKV579 | [H]TRQVMSMVSSWLWKE[OH] |  | ZIKV640 | [H]FAHALRFLNDMGKVR[OH] |  |  |  |
| ZIKV580 | [H]SMVSSWLWKELGKHK[OH] |  | ZIKV641 | [H]RFLNDMGKVRKDTQE[OH] |  |  |  |
| ZIKV581 | [H]WLWKELGKHKRPRVC[OH] |  | ZIKV642 | [H]MGKVRKDTQEWKPST[OH] |  |  |  |
| ZIKV582 | [H]LGKHKRPRVCTKEEF[OH] |  | ZIKV643 | [H]KDTQEWKPSTGWDNW[OH] |  |  |  |
| ZIKV583 | [H]RPRVCTKEEFINKVR[OH] |  | ZIKV644 | [H]WKPSTGWDNWEEVPF[OH] |  |  |  |
| ZIKV584 | [H]TKEEFINKVRSNAAL[OH] |  | ZIKV645 | [H]GWDNWEEVPFCSHHF[OH] |  |  |  |
| ZIKV585 | [H]INKVRSNAALGAIFE[OH] |  | ZIKV646 | [H]EEVPFCSHHFNKLHL[OH] |  |  |  |
| ZIKV586 | [H]SNAALGAIFEEEKEW[OH] |  | ZIKV647 | [H]CSHHFNKLHLKDGRS[OH] |  |  |  |
| ZIKV587 | [H]GAIFEEEKEWKTAVE[OH] |  | ZIKV648 | [H]NKLHLKDGRSIVVPC[OH] |  |  |  |
| ZIKV588 | [H]EEKEWKTAVEAVNDP[OH] |  | ZIKV649 | [H]KDGRSIVVPCRHQDE[OH] |  |  |  |
| ZIKV589 | [H]KTAVEAVNDPRFWAL[OH] |  | ZIKV650 | [H]IVVPCRHQDELIGRA[OH] |  |  |  |
| ZIKV590 | [H]AVNDPRFWALVDKER[OH] |  | ZIKV651 | [H]RHQDELIGRARVSPG[OH] |  |  |  |
| ZIKV591 | [H]RFWALVDKEREHHLR[OH] |  | ZIKV652 | [H]LIGRARVSPGAGWSI[OH] |  |  |  |
| ZIKV592 | [H]VDKEREHHLRGECQS[OH] |  | ZIKV653 | [H]RVSPGAGWSIRETAC[OH] |  |  |  |
| ZIKV593 | [H]EHHLRGECQSCVYNM[OH] |  | ZIKV654 | [H]AGWSIRETACLAKSY[OH] |  |  |  |
| ZIKV594 | [H]GECQSCVYNMMGKRE[OH] |  | ZIKV655 | [H]RETACLAKSYAQMWQ[OH] |  |  |  |
| ZIKV595 | [H]CVYNMMGKREKKQGE[OH] |  | ZIKV656 | [H]LAKSYAQMWQLLYFH[OH] |  |  |  |
| ZIKV596 | [H]MGKREKKQGEFGKAK[OH] |  | ZIKV657 | [H]AQMWQLLYFHRRDLR[OH] |  |  |  |
| ZIKV597 | [H]KKQGEFGKAKGSRAI[OH] |  | ZIKV658 | [H]LLYFHRRDLRLMANA[OH] |  |  |  |
| ZIKV598 | [H]FGKAKGSRAIWYMWL[OH] |  | ZIKV659 | [H]RRDLRLMANAICSSV[OH] |  |  |  |
| ZIKV599 | [H]GSRAIWYMWLGARFL[OH] |  | ZIKV660 | [H]LMANAICSSVPVDWV[OH] |  |  |  |
| ZIKV600 | [H]WYMWLGARFLEFEAL[OH] |  | ZIKV661 | [H]ICSSVPVDWVPTGRT[OH] |  |  |  |
| ZIKV601 | [H]GARFLEFEALGFLNE[OH] |  | ZIKV662 | [H]PVDWVPTGRTTWSIH[OH] |  |  |  |
| ZIKV602 | [H]EFEALGFLNEDHWMG[OH] |  | ZIKV663 | [H]PTGRTTWSIHGKGEW[OH] |  |  |  |
| ZIKV603 | [H]GFLNEDHWMGRENSG[OH] |  | ZIKV664 | [H]TWSIHGKGEWMTTED[OH] |  |  |  |
| ZIKV604 | [H]DHWMGRENSGGGVEG[OH] |  | ZIKV665 | [H]GKGEWMTTEDMLVVW[OH] |  |  |  |
| ZIKV605 | [H]RENSGGGVEGLGLQR[OH] |  | ZIKV666 | [H]MTTEDMLVVWNRVWI[OH] |  |  |  |
| ZIKV606 | [H]GGVEGLGLQRLGYVL[OH] |  | ZIKV667 | [H]MLVVWNRVWIEENDH[OH] |  |  |  |
| ZIKV607 | [H]LGLQRLGYVLEEMSR[OH] |  | ZIKV668 | [H]NRVWIEENDHMEDKT[OH] |  |  |  |
| ZIKV608 | [H]LGYVLEEMSRIPGGR[OH] |  | ZIKV669 | [H]EENDHMEDKTPVTKW[OH] |  |  |  |
| ZIKV609 | [H]EEMSRIPGGRMYADD[OH] |  | ZIKV670 | [H]MEDKTPVTKWTDIPY[OH] |  |  |  |
| ZIKV610 | [H]IPGGRMYADDTAGWD[OH] |  | ZIKV671 | [H]PVTKWTDIPYLGKRE[OH] |  |  |  |
