# Supplementary material for: CD4+T cells mediate protection against Zika associated severe disease in a mouse model of infection
Source: PLoS Pathog. 2018 Sep 13;14(9):e1007237. doi: 10.1371/journal.ppat.1007237 (PMC6136803; doi:10.1371/journal.ppat.1007237)
Supplement: S2 Table — Amino acid residues at 15-mer loci PrM251, E646, NS1811, and NS53211 from different strains of ZIKV were compared to that of the reference library (PRVABC59). Three strains of Asian lineage were compared including R103451 (GenBank:KX694534), P6-740 (GenBank:KX377336), and FLR (GenBank:KU820897). Three strains of African lineage were also compared including MR766 (GenBank:KX377335), DAK AR (GenBank:KY348860), and IbH (GenBank:KU963574). Residues that differ from the reference sequence for the library (PRVABC59) are highlighted in grey and written in red. (DOCX) [file ppat.1007237.s004.docx]

| **Lineage** | **Strain** | **PrM_251_** | **E_646_** | **NS1_811_** | **NS5_3211_** |
| --- | --- | --- | --- | --- | --- |
| **Asian** | **PRVABC59** | IFRNPGFALAAAAIA | GRLITANPVITESTE | TGVFVYNDVEAWRDR | KDTQEWKPSTGWDNW |
|  | **R103451** | IFRNPGFALAAAAIA | GRLITANPVITESTE | TGVFVYNDVEAWRDR | KDTQEWKPSTGWDNW |
|  | **P6-740** | IFRNPGFALAAAAIA | GRLITANPVITESTE | TGVFVYNDVEAWRDR | KDTQEWKPSTGWSNW |
|  | **FLR** | IFRNPGFALAAAAIA | GRLITANPVITESTE | TGVFVYNDVEAWRDR | KDTQEWKPSTGWDNW |
| **African** | **MR766** | IFRNPGFALVAVAIA | GRLITANPVITESTE | TGVFIYNDVEAWRDR | KDTQEWKPSTGWSNW |
|  | **DAK AR** | IFRNPGFALVAVAIA | GRLITANPVITESTE | TGVFVYNDVEAWRDR | KDTQEWKPSTGWSNW |
|  | **IbH** | IFRNPGFALVAVAIA | GRLITANPVITESTE | TGVFIYNDVEAWRDR | KDTQEWKPSTGWSNW |
